# Abiotic Stress and Belowground Microbiome: The Potential of Omics Approaches

**DOI:** 10.3390/ijms23031091

**Published:** 2022-01-19

**Authors:** Marco Sandrini, Luca Nerva, Fabiano Sillo, Raffaella Balestrini, Walter Chitarra, Elisa Zampieri

**Affiliations:** 1 Research Center for Viticulture and Enology, Council for Agricultural Research and Economics, 31015 Conegliano, Italy; marco.sandrini@crea.gov.it (M.S.); luca.nerva@crea.gov.it (L.N.); walter.chitarra@crea.gov.it (W.C.); 2 National Research Council, Institute for Sustainable Plant Protection, Strada delle Cacce 73, 10135 Torino, Italy; fabiano.sillo@ipsp.cnr.it (F.S.); elisa.zampieri@ipsp.cnr.it (E.Z.)

**Keywords:** climate change, abiotic stress, omics tools, arbuscular mycorrhizal fungi, Actinomycetes, plant–microbe interactions

## Abstract

Nowadays, the worldwide agriculture is experiencing a transition process toward more sustainable production, which requires the reduction of chemical inputs and the preservation of microbiomes’ richness and biodiversity. Plants are no longer considered as standalone entities, and the future of agriculture should be grounded on the study of plant-associated microorganisms and all their potentiality. Moreover, due to the climate change scenario and the resulting rising incidence of abiotic stresses, an innovative and environmentally friendly technique in agroecosystem management is required to support plants in facing hostile environments. Plant-associated microorganisms have shown a great attitude as a promising tool to improve agriculture sustainability and to deal with harsh environments. Several studies were carried out in recent years looking for some beneficial plant-associated microbes and, on the basis of them, it is evident that Actinomycetes and arbuscular mycorrhizal fungi (AMF) have shown a considerable number of positive effects on plants’ fitness and health. Given the potential of these microorganisms and the effects of climate change, this review will be focused on their ability to support the plant during the interaction with abiotic stresses and on multi-omics techniques which can support researchers in unearthing the hidden world of plant–microbiome interactions. These associated microorganisms can increase plants’ endurance of abiotic stresses through several mechanisms, such as growth-promoting traits or priming-mediated stress tolerance. Using a multi-omics approach, it will be possible to deepen these mechanisms and the dynamic of belowground microbiomes, gaining fundamental information to exploit them as staunch allies and innovative weapons against crop abiotic enemies threatening crops in the ongoing global climate change context.

## 1. Introduction

The association between plants and microorganisms is a well-known concept whose importance has already been demonstrated in several experimental works [1]. Plants cohabit with a plethora of microorganisms both inside and outside their tissues [2]. Nowadays, plants and their associated microorganisms are considered as unique organisms called “holobionts”, and the research is focused on deciphering the interactions occurring among them and the resulting impact on plant fitness [3]. It is not wrong therefore to claim that plant wellness is grounded on ecological interactions with its own microbiome, and this is reflected in the unfeasibility to obtain “gnotobiotic” plants, i.e., without associated microorganisms, which immediately appear to be suffering and quickly die [4]. These microorganisms are involved in major plant functions such as nutrition and tolerance/resistance to abiotic and biotic stresses, improving growth and modulating and complementing defense responses [5].

However, agriculture is also characterized by a large use of chemical inputs to improve crop productivity and to control plant pathogens. Thus, the soil microbial biodiversity is often subjected to a negative selection pressure, which leads to its weakening and decline [6]. Modern agriculture is now entering a new ‘green revolution’ and microbial biodiversity importance is rising. In this context, new environmentally friendly management strategies are needed to enhance agriculture sustainability and to preserve the microbiome richness and biodiversity. Moreover, the ongoing climate change scenario has been threating agriculture and food security, increasingly inducing plant abiotic stresses. According to an estimation by Mahajan and colleagues [7], abiotic stress factors can cause a reduction in average yields for major crops by more than 50%. Plant-associated microorganisms seem to be a very promising tool for facing this negative scenario and they can be considered as an interesting ally of modern agriculture in dealing with climate change. Chialva et al. [8] applied RNA-seq and proteomics, in addition to a direct quantification of biochemical compounds, to roots of plants grown in native soils. Results showed that native soils, in contrast to disinfected ones, elicited an alert status in the plant by enhancing the induction of genes involved in defense responses. Indeed, a disease-suppressive soil was more effective in priming resistance compared to disinfected substrates, supporting the hypothesis that microbiota contained in different soils may trigger different plant responses. Beyond this background, researchers are looking for new sustainable strategies for field management by improving microbial biodiversity, favoring the beneficial ones and reducing the accumulation of phytopathogenic species [9]. Two of the most promising actors playing a crucial role in the race between abiotic stresses and plants are represented by arbuscular mycorrhizal fungi (AMF) and Actinomycetes. AMF can improve the plants’ capacity to deal with pathogen infections and they can also be considered as biofertilizers [10]. Mycorrhizal fungi absorb phosphate, nitrogen, and other macronutrients, microelements, and water from the soil and then they provide these resources to the host plant [11], while the host plant provides carbon compounds such as carbohydrates and lipids to fungus [12]. The most common mycorrhizal association is made up by the AMF that interact with the roots of most terrestrial plants [12]. AMF belong to the subphylum Glomeromycotina [13] and they are obligate biotrophs. Conversely, Actinomycetes are beneficial bacteria that interact with plants as free-living non-obligated symbiotic organisms, and they have shown over time great potential in improving the future of agriculture [14]. They have been found mainly in the soil, but they are also able to colonize plant roots and tissue, living as endophyte or establishing a mutualistic relationship with plants. The Actinomycetes are aerobic Gram-positive bacteria, belonging to the order Actinomycetales within the phylum Actinobacteria containing genera of filamentous bacteria [15]. These filamentous Actinomycetes resemble fungi in their morphology, forming branching hyphae, asexual spores, and mycelium [14]. Actinomycetes are ubiquitous organisms, mostly saprophytes and chemoorganotrophic, able to degrade polymers, i.e., chitin and cellulose, and complex macromolecules such as lignocellulose, xylan, and lignin, which afford them a nutritional advantage [14]. They can support plants via several mechanisms, such as solubilizing phosphate, fixing nitrogen, and producing ammonia [16,17,18].

This review is therefore focused on these beneficial organisms and on their capacity to support plants under harsh environmental conditions, highlighting the role of omics tools to elucidate the mechanisms involved in plant–microbe interactions. Limitations and caveats in the use of these approaches, as well as the importance to combine them with biochemical and physiological data to obtain information on mechanisms involved in the interactions, will also be underlined.

## 2. Plant Responses to Abiotic Stresses

Plants are continuously exposed to environmental stresses affecting their growth and yield, both biotic such as pest and pathogens, and abiotic such as heat, cold, drought, salinity, waterlogging, heavy metal toxicity, nutrient deficiency, and oxidative stresses [19]. Climate change has been amplifying the incidence of hostile events that can determine several negative impacts [20]. The devastating effects of soil pollution and extreme climatic events are becoming commonplace, and the linked abiotic stresses affecting crops are gaining more importance in agriculture every year. There are different types of abiotic stresses [20], and among these, drought and salinity represent the major environmental factors limiting crop production [21]. Drought is becoming increasingly important because of the rising temperature and the intensifying desertification processes, mainly in arid and semiarid regions. On the other hand, soil salinity, as a result of the deposition of salt after either natural (primary) or anthropogenic (secondary) processes, is a rising threat to agricultural soils. Primary processes are associated with the weathering of parent rocks, seawater, or atmospheric deposition. Secondary processes embrace poor drainage facilities, irrigation with brackish groundwater, nonstop irrigation for long durations, unsuitable water management, and cultural methods in irrigated agriculture [22]. 

In relation to the timing, duration, and intensity of abiotic stress, plant susceptibility changes. Stress determines changes in numerous physiological, biochemical, and molecular processes [20,23]. For instance, high salt deposition in the soil causes osmotic and ionic stresses, and then oxidative stress in plants [22]. To respond to the ever-changing environments, plants have a defense system that can be enhanced and alerted using biological and chemical priming, thus making them “ready for the battle” [24]. Priming (or acclimatization) is a complex phenomenon that consists in preconditioning the plant immune system and abiotic defenses, making the plant responses to stress quicker, stronger, and more effective [20,24]. It starts with stress sensing or perception by sensors, which are molecules or structures that change shape or temporarily lose their function, initiating a signaling cascade that causes a response [25]. These sensors determine reversible physical changes (e.g., the change in membrane fluidity and in protein conformation, partial separation, or melting of DNA and RNA strands), causing a differential transcription control and stress-responsive gene regulation [20]. Receptors can belong to G Protein-Coupled Receptors (GPCRs), where the ligand presence causes conformational changes and helps the exchange of GTP for GDP, which then allows the activation of heterotrimeric guanine-nucleotide-binding proteins (G proteins) [20]. Receptor-Like Kinases (RLKs) and Histidine Kinases (Hks) are also known to be receptors to sensors [20]. The first site of stress sensing is represented by the cell surface or cell membrane, and this causes fluctuations of the cytosolic calcium level. Ca^2+^ is a secondary stress messenger, which transmits the stress signals from cell surface/membrane receptors to effector proteins, activating other messengers/sensors such as calcineurin B-like proteins (CBLs), calmodulin (CaMs), calmodulin-like proteins (CMLs), and Calcium-Dependent Protein Kinases (CDPKs/CPKs) [20]. Another signaling cascade is represented by Mitogen-Activated Protein Kinase (MAPK) that transmits a signal via phosphorylation and includes MAPKK kinases (MAPKKKs, MAP3K, or MEKK), MAPK kinases (MAPKKs, MAP2Ks, MKKs, or MEKs), and MAPKs (MPKs) [20].

The plant defense system can be stimulated by beneficial microbes, chemical compounds, insects, or environmental signals [26]. Plant biostimulants can therefore be considered key emerging strategies for improving crop production and resilience to climate change [23]. In the presence of abiotic stresses, one of the first plant responses is the phytohormone production (abscisic acid (ABA), auxins or indole acetic acid (IAA), ethylene (ET), cytokinins (CK), gibberellins (GA), salicylic acid (SA), jasmonic acid (JA), and brassinosteroids (BRs)), which act as primary signaling molecules that lead to the expression of stress-related genes (e.g., accumulation of reactive oxygen species (ROS) and antioxidant enzymes) and the induction of physiological and morphological changes ([23] and references therein). The physiological responses are characterized by a reduction in transpiration, photosynthesis, and growth rates, and a decrease in stomatal conductance and in leaf water content ([23] and references therein). A biochemical response is the perturbation of ROS homeostasis, causing accumulation of singlet oxygen, superoxide anion radical, hydrogen peroxide, and hydroxyl radical [27]. Plants can manage this oxidative stress by deploying a scavenging system based on both enzymes, such as superoxide dismutase (SOD), ascorbate peroxidase (APX), catalase (CAT), glutathione peroxidase (GPX), peroxidase (POX), and glutathione reductase (GR), and a non-enzymatic antioxidant mechanism, made-up of low molecular weight compounds, e.g., phenolic compounds, amino acids, carotenoids, glutathione (GSH), ascorbic acid, and α-tocopherol [28]. In drought conditions, for example, shoot growth inhibition, as well as the biosynthesis and assimilation of metabolites such as low molecular weight osmolytes and soluble compounds with a key role in keeping osmotic balance and in stabilizing membranes and macromolecules, has been documented. Macromolecules include betaines, amino acids, polyols, glycine betaine, proline, and inositol [29].

## 3. Role of Soil Microbes in Interaction with Plants under Abiotic Stress Conditions

Plants are not standalone entities, and under natural conditions they do not live alone, in a sterile environment, but they are in a close relationship with several microorganisms, such as bacteria, fungi, protists, viruses, etc. [30]. These associated microorganisms cohabit in different plant tissues, constituting the plant microbiome, which is the microbial community living in three different habitats: phyllosphere (stem and leaf surfaces), endosphere (internal tissues within the plant), and rhizosphere (root surfaces and the soil around them) [2].

It is increasingly evident that both beneficial soil bacteria, such as plant growth-promoting bacteria (PGPB) and rhizobia, and fungi, such as mycorrhizal fungi, have a great potential in sustainable agriculture, improving plant growth and enhancing plant tolerance to abiotic stresses [31]. Here, we will focus on the interactions with two groups of microorganisms that are receiving greater attention for the potential in stressed environments, i.e., the mutualistic symbiosis formed by AMF and the associations with Actinomycetes, underlining the role of these interactions in mitigating the impact of environmental stresses.

### 3.1. Arbuscular Mycorrhizal Fungi

AMF are symbiotic soil-borne fungi that can impact plant development and health [32]. Although they are considered nowadays to have a great potential for sustainability and stability in agricultural production and great progress has been made in understanding the AM symbiosis, considerable gaps of knowledge are still to be filled in optimizing agroecosystem services associated with AMF. In the literature, there is also evidence of the protective role of AMF in the presence of abiotic stresses when multiple stresses are in combination ([33] and references therein) and specifically when AMF are in a relationship with tomato roots, considering that, mainly in the last year, several works have been focused on this crop ([34] and references therein). AMF can protect from salt stress both in relationship with halophytes and glycophytes [35]. Recently, Pan et al. [36] demonstrated on the contrary a greater dependence of glycophytes vs. AMF than of halophytes in the presence of salt stress. Two meta-analyses tried to decipher the benefits of AMF during salt stress [37,38]. AMF can increase the osmolytes, carbohydrates, and antioxidant systems [39]. When they are in symbiosis with plants, a high K^+^/Na^+^ ratio is maintained, avoiding uptake or translocation of toxic Na^+^ [40]. AMF can also influence carbon use efficiency, maintaining a higher grain yield, a higher rate of net photosynthesis and stomatal conductance, and lower intrinsic water use efficiency under salt stress [41]. Recently, Evelin et al. [22] discussed AMF-mediated mechanisms from nutrient uptake to water use efficiency improvement, from ionic homeostasis maintenance and osmoprotection to improved photosynthetic efficiency and photosynthetic apparatus defense, and from cell ultrastructure protection to strengthened and induced antioxidant metabolism, until the modulation of the phytohormone profile ([22] and references therein). In the giant reed *Arundo donax*, the negative effect of salt on plant performance was not rescued by AM fungal inoculation, although modifications in plant metabolism were observed in AM-colonized plants, such as a significant increase in proline level and a trend toward higher isoprene emission and higher H_2_O_2_ in comparison with not-inoculated plants [42].

AMF also have a positive effect on tomato plants in the presence of water stress, but the effects changed in function of considered traits and fungal species [43,44]. Studying the effect of three AMF of different genera on tomato tolerance to drought or salt stress, it was evident that some responses were common to all tested AMF, while others were specifically related to single isolates [45]. Moreover, AMF have positive effects on maize under drought conditions, favoring plant growth and photosynthesis by significantly improving chlorophyll content, mineral uptake and assimilation, compatible solute content, and inducing the antioxidant system [46]. It has also been demonstrated that AMF reduce damage to the photosystems PSII and PSI structures and functions under water stress [47,48]. Recently, Zou et al. [49] suggested that the protective role against ROS burst was based on the activation of fungal genes coding for scavenging enzymes such as SOD and non-enzymatic defenses such as metallothionein, glutaredoxin, etc. ([49] and references therein). There are, on the contrary, contrasting results concerning the effects of AMF on plant hormone content and on aquaporin expression because they depend on the fungus, type of aquaporin, and the imposed stress ([50] and references therein). Considering that dryness and salinity induce similar overall stresses (water deficit condition), it is likely that AMF, which allow the plants’ survival in high salinity conditions, may also make plants tolerant to drought [50].

Transcriptomics provided useful information on the plant and fungal gene regulation during AM symbiosis in diverse crop species such as rice [51,52], tomato [53], grapevine [54], and wheat [55]. Transcriptomics has been recently applied in AM-colonized tomato roots to verify the role of AM symbiosis in tomato response to water stress and nematode infection [56]. Results provided novel information about the response to AM symbiosis, showing both tomato and fungal genes involved in water stress response during symbiosis. Additionally, changes in the expression of tomato genes related to nematode infection during AM symbiosis suggested a role of AM colonization in triggering defense responses against root-knot nematodes [56].

However, notwithstanding the several studies that have already been conducted on the impact of AM fungal colonization on plant response to abiotic stresses, information on AM-mediated photosynthesis mechanisms and on the role of the AM symbiosis in nutrient use efficiency in stress conditions is still patchy and limited with respect to the response of different plant genotypes [47].

### 3.2. Actinomycetes

Many reports have documented that Actinomycetes can grow under diverse stress conditions such as drought, high temperature, and high salinity, and they have an important role in alleviating harmful damages caused by abiotic stresses promoting plant growth [57]. Plant growth promotion traits combined with the harsh environmental condition tolerance potential in the Actinobacteria play a synergistic role, supporting enhanced plant growth promotion [58]. Moreover, thanks to their peculiar morphology, they cleave to the rhizospheric soil particles, forming a strong bond with the plants. This attitude allows a more efficient use of nutrients and water in the rhizosphere and supports the plant in stressed soil. Improving tolerance to abiotic stresses by these bacteria acts through several mechanisms, ranging from phytohormonal modifications and 1-aminocyclopropane-1-carboxylic acid (ACC) deaminase activity, to alterations in root and cell wall morphology, as well as in the capability to avoid oxidative damage and the compatible solutes’ production (glycine-betaine and proline) that assist in the processes of osmoregulation [17]. Aly and colleagues [59] have observed that soaking wheat seeds in *Streptomyces* sp. significantly increased their germination in high-salinity conditions, and soil inoculations with *Streptomyces* bacteria significantly improved root depth, shoot length, and shoot and root dry weights. Furthermore, Palaniyandi et al. [60] demonstrated that *Streptomyces* sp. PGPA39-inoculated tomato plants showed a significant increase in plant biomass and chlorophyll content and a reduction in leaf proline content under salt stress compared to the control and salt-stressed non-inoculated plants. It has also been demonstrated that Actinobacteria are characterized by the ability to promote wheat growth in water stress conditions [59]. Maize plants in drought conditions inoculated with Actinomycetes grew better than those that were un-inoculated, and they also showed better survival, dry root and shoot weight, root and shoot length, and chlorophyll content [17]. Additionally, Hasegawa and colleagues [61] have shown that endophytic *S. pada* AOK-30 might increase drought tolerance in mountain laurel (*Kalmia latifolia* L.), inducing cell wall structural modification with callose accumulation and lignification. Thanks to the next-generation sequencing (NGS) technologies of microbial communities, Actinobacteria have frequently been found to be one of the five most dominant bacterial phyla in soils [62,63,64]. However, until now, many studies about Actinomycetes and their important role in alleviating abiotic plant stresses have been performed, but the interaction dynamic between them and plants is still largely unknown, limiting the possibility to exploit these microorganisms in agriculture. Omics approaches seem to be a very promising way to support the research in unearthing this hidden world and the mechanisms on which this fundamental cooperation is based on.

## 4. Diversity and Complementarity of Omics Approaches in Studying Plant–Microbe Interactions

The progress in sequencing technologies and several omics tools have allowed to better decipher plant–microbe interactions [2], both in terms of biodiversity and gene expression regulation. The microbial community colonizing the rhizosphere and the surrounding soil is known to affect the plant resistance against abiotic stresses, and microbiome-based multi-omics studies have shown the potential to significantly advance the knowledge of rhizospheric science, allowing the characterization of plant-associated beneficial microorganisms and their functions [65]. For instance, next-generation sequencing (NGS) on DNA extracted from soil and rhizosphere have been used to infer their microbial communities, providing novel ways to capitalize on plant-associated microorganisms. This approach has led to a better understanding of the structure, abundance, spatial distribution diversity, and important members of the rhizosphere community [66]. A challenge in the analyses of omics data is the choice of bioinformatics tools to deal with large sequenced datasets with minimal errors, in a fast and accurate way and with huge data storage [67]. Nowadays, the increasing omics, bioinformatics, and statistical tools would allow to produce robust predictions and models based on crop–soil–climate data about plant adaptation under climate change [68,69]. Furthermore, the data originated from the large-scale analyses lead to the possible identification of functional genes that might be subsequently manipulated to obtain climate-resistant varieties and crops for a sustainable agriculture [70], i.e., through the recent developed genome editing approaches. Among them, the clustered regularly interspaced short palindromic repeats (CRISPR/Cas9) technology, which can be used to produce knockout non-transgenic plant and microbe mutants, is nowadays very “exploited”. Such approach could be useful both to characterize symbiosis-related proteins and plant traits that sustain beneficial microbiomes and to identify key genes or genetic factors for stress tolerance, and to assign them a specific function [71,72].

**Figure 1:**
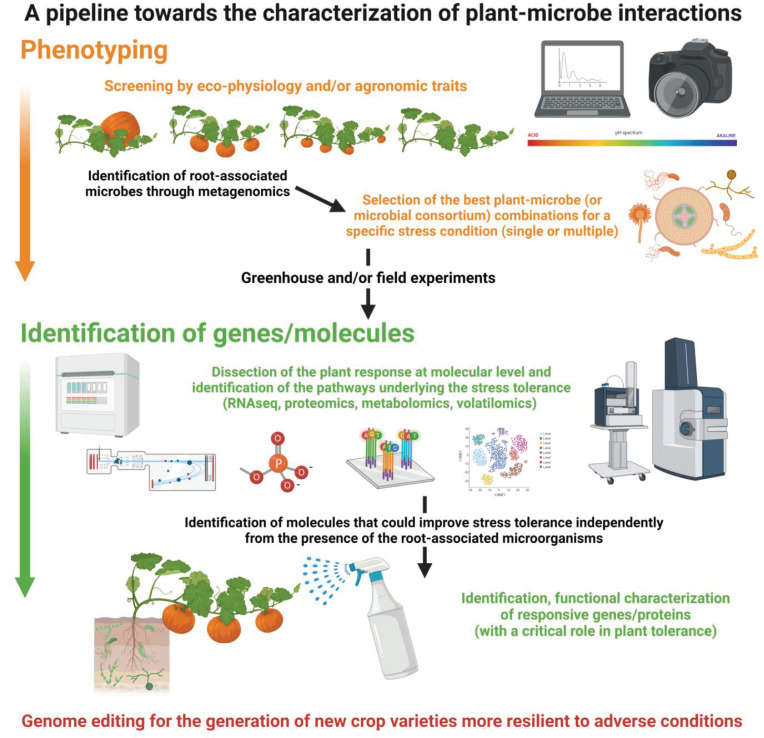
The flow-chart represents a pipeline to study plant–microbe interactions for obtaining crop varieties more tolerant and resilient to environmental stresses.

Obviously, each omics approach shows limits that may influence the sensitivity or specificity of the technique itself, but by combining different approaches, it could be possible to overcome some limitations. Different approaches in fact provide the opportunity to answer to different questions: Who is present? What is it doing? They can be defined by their molecular targets, i.e., DNA, RNA, proteins, or metabolites, etc. The diverse omics approaches, i.e., genomics, transcriptomics, proteomics, and metabolomics, provide useful information to explain plant-microbe relationships in terms of the underlying molecules and their interactions. Then, the task is to convert molecular catalogs of molecules (genes, proteins, metabolites) into a dynamic understanding of symbiosis function ([73], Supplementary Tables S1 and S2). Bioinformatics progress has already allowed to better decode data and to integrate results from different omics techniques [74], providing a more complete picture of plant microbiota and their interaction with the host. Considering the differences among the outputs of the diverse techniques, the possibility to use combined approaches in an experiment is a relevant point. The possibility to screen a large number of genotypes, also looking at the root-associated microbes through a metagenomics approach, in diverse environments can be a relevant point to select the best microbial communities in terms of stress tolerance. Then, the application of the other omics approaches, such as transcriptomics and metabolomics, leads to understanding the mechanisms involved in the interactions as well as in the improving tolerance (Figure 1). This approach could also lead to the identification of novel molecules that may improve stress tolerance independently from the presence of root-associated microorganisms. Relevant molecules promoted by the symbiosis or by the presence of specific root-associated microorganisms could be applied to plants that do not form symbiosis with root-symbiotic microbes as well as in the absence of the microbial species that produce a specific effective molecule. Additionally, it is worth noting that the integration with physiological data (e.g., photosynthetic rate, stomatal conductance) and biochemical analyses on stress-related molecules (e.g., proline, H_2_O_2_) and enzymes (e.g., superoxide dismutase) is a required step to obtain mechanistic and functional insights. 

### 4.1. Metagenomics vs. Metabarcoding

Thanks to next-generation DNA sequencing methods (e.g., 454 pyrosequencing) and second- and third-generation sequencing platforms (e.g., Illumina MiSeq, HiSeq, NovaSeq, Ion Torrent PGM GeneStudio, PacBio RSII Sequel, Oxford Nanopore MinION, GridION, and PrometION) [75], plant–microbe complexity has been explored. To identify microbial groups and to compare them on the basis of richness, evenness, composition, and assembly [2], two main approaches can be adopted: shotgun metagenomics and metabarcoding [76]. Metagenomics, defined as the genomic analysis of organisms in environmental samples, offers information about taxonomic composition and relative abundance [77,78]. It identifies the genomic diversity and gene function of microbes, going beyond traditional genomic techniques [79]. A classic metagenomics pipeline involves the isolation of environmental DNA and library preparation and sequencing, followed by the bioinformatics pre-processing of DNA sequence reads that allows to define the taxonomic profile and functional or genomic elements. Further steps include statistical assessment of data, data validation, and lastly, visualization and communication of the results [80]. In metabarcoding, a single marker gene is amplified from environmental DNA, such as the 16S rRNA gene for prokaryotes and the internal transcribed spacer (ITS) or the large ribosomal subunit (LSU) for eukaryotes. The amplified sequences generally allow genera or species identification [81]. Since the first studies where 454 pyrosequencing was applied to study AMF community diversity in soil and roots [82,83], metagenomics/metabarcoding approaches have been widely used, also thanks to the development of primers suitable for the identification of this group of fungi, to verify the AMF communities and their shifts in diverse ecosystems [84]. The success of metabarcoding is strictly associated to its limited costs for obtaining huge data, enabling to decipher diversity. However, some important biases may affect its outcomes. These biases include PCR-derived sequence errors, possibly causing overestimation of biodiversity and insufficient resolution for taxonomic assignment due to primer choice [85]. Metabarcoding may in fact be affected by PCR primer amplification efficiency and copy number variation of the marker gene (e.g., ITS) in different species, thus meaning that not all species present in the samples have the same chance to be detected [86]. On the other hand, the metagenomics approach provides not only taxonomic identification, but also functional information. However, the sequencing depth has a significant impact on the results [85].

A major challenge in metagenomics/metabarcoding is how to clearly state the microbial species name. Different databases such as GenBank, Greengenes (for bacteria), and UNITE (for fungi) are available, but they all suffer from a lack of genomic information about the huge microbe variety still undiscovered on the earth [87]. Moreover, both dead and living organisms are detected by metagenomics/metabarcoding, not allowing to distinguish contaminant DNA from DNA of the living microbial community [88], differently from the meta-transcriptomics approach.

### 4.2. Meta-Transcriptomics and Meta-Proteomics

Meta-transcriptomics and meta-proteomics provide functional data, clarifying what is happening in a complex matrix such as a specific soil sample. The first is focused on the evaluation of expressed genes [75], while the second on the study of all the proteins present in a biomass [89,90]. The sequencing of transcripts (the so-called RNA-seq) can allow to carry out ecological studies of microbial communities [91]. A major constraint could be the difficulty in interpreting RNA-seq results, for instance without the availability of a well-annotated reference genome to be used for RNA-seq reads’ mapping. However, the growing availability of annotated transcriptomes in curated databases, as well as the development of a robust de novo RNA-seq assembler, may help in result explanation [88]. Recently, Chialva et al. [92] have been able to reconstruct the microbial communities and obtain an overview of their functional diversity using a previously generated RNA-seq dataset for tomato roots collected from plants growing on different native soils. Meta-proteomics allows to characterize biological processes and metabolic pathways, to understand plant–microbe interaction functions, structures, dynamics, significance, molecular basis of cell communication, and symbiotic development regulation [72]. Unfortunately, it is impeded both by technical and computational limitations, such as low protein quality and concentration, contaminants interfering with the protein extraction, and the absence of complete protein databases [71,93,94,95]. One of the major limitations could be represented by both the incompleteness of databases (taxonomic, related to chemical compounds or proteins) and by the absence of standardized metadata [93], even if curated open-source databases are already established (e.g., UNITE [96], PRIDE [97]).

### 4.3. Metabolomics

Metabolomics is the study of all small molecules within an organism which are produced on the basis of the information cascade contained in the genome, and transcribed and translated in the transcriptome and proteome, respectively [98]. Current metabolomics technology is based on ultra-high-pressure liquid chromatography coupled with high-resolution mass spectrometry or nuclear magnetic resonance spectroscopy, and also provides a chemical profile of thousands of compounds [87]. It can be targeted (i.e., comparison with a known set of annotated compounds) [99] or untargeted (i.e., comparison on the basis of relative intensities) [100]. A key caveat in metabolomics is the huge variety of potential metabolites present in any given sample [87] and the limited extents of public metabolite reference databases, thus making it difficult to assign a measured metabolite to a specific organism [71]. Untargeted metabolomics approaches were already used to obtain novel information on the impact of AM symbiosis. Changes in metabolomics profile have been showed in mycorrhizal roots [45,101], allowing to identify potential primed compounds putatively involved in the enhanced stress tolerance observed in mycorrhizal plants [45,102,103]. More recently, an untargeted metabolomics analysis through UPLC-MS was performed on mycorrhizal plant leaves. The results showed that mycorrhizal symbiosis had a very limited impact on the leaf metabolome in the absence of stress, while it significantly modulated the plant response to herbivory in the damaged area, where a local accumulation of defense compounds was observed [104]. Metabolomics is also essential to study exudates released by the roots in the rhizosphere, which constitute a fundamental feed source for the microorganisms associated with the roots compared to the surrounding soil that is poorer in nutrients [105]. Interestingly, they can vary according to the age and developmental stages of plants, to different plant species, but even among different genotypes (therefore different varieties) within the same species, e.g., during domestication [106].

### 4.4. Non-Destructive Approaches (Phenomics)

The selection of phenotypes is at the basis of the breeding process. Nowadays, high-throughput plant phenotyping records complex traits related to growth, yield, and adaptation to stress, with an improved accuracy and precision at different scales of organization, from organs to canopies, including the basic measurement of individual quantitative parameters that underlie complex trait assessment [107,108,109]. It is founded on non-destructive image analysis, data management, and modeling, and it is emerging as a cutting-edge technology with a relevant role in plant and agronomic sciences to design new crops and to characterize the genetic response to environmental stimuli, thus improving the breeding and management of crops [110]. The plant phenotype derives from the interaction with the spatially and temporally dynamic environment above and below ground [108]. Core points of modern phenotyping are: (i) non-destructive measurements that allow to follow a trait over time, and (ii) high-throughput measurements, by which screening several species and genotypes at similar conditions is possible [109]. Phenotyping leads to the quantification of diverse structural and functional aspects, such as plant biomass, root morphology and architecture, leaf characteristics, fruit traits, photosynthetic efficiency, and biotic and abiotic stress resistance/tolerance, but also chemical phenotypes, such as secondary metabolites with roles in plant defense and the interaction with the environment, i.e., the emission of volatile organic compounds (VOCs) [109]. A further step is the development of protocols that allow to verify the impact of root-associated microbes on a large number of species and genotypes contemporaneously, with the aim to identify the best plant–microbe combinations in terms of plant performance and resilience.

#### 4.4.1. Volatilomics

Volatilomics is a field of metabolomics focusing on the detection, characterization, and quantification of compounds released by an organism, i.e., VOCs, which allow the communication between organisms, playing an important role in the development and regulation of symbiotic interactions [111] and acting in the plant defense response against both abiotic and biotic stresses [44,112,113]. It is worth noting that plant microbiota, in addition to having an impact on the plant emission, produces VOCs too, which can elicit plant defenses and inhibit the growth and development of plant pathogens [114,115]. Nowadays, these molecules have achieved resounding success since, once identified and quantified, they could be extracted and used as biostimulants in sustainable agriculture [116]. The approach is based on gas chromatography coupled with mass spectrometry (GC-MS), where the critical point is the choice of VOC collection method [87]. VOCs’ detection could also be obtained using an Ultra-Sensitive High-Resolution PTR-QiTOF [117]. However, although this approach enables the detection of different VOC emission blends, the precise identification of specific VOCs should be confirmed by GC-MS.

#### 4.4.2. Shovelomics

The root system has many major functions in water and nutrients supply, acting as food and anchoring the plant to the soil ([118] and references therein). Root architecture refers to the spatial arrangement of the root system in the soil [119] and several other features, such as root length, primary root branch, lateral root branch, density, diameter, angle, and total root surface, defining the whole root system architecture [118,120]. Root systems characterized by different root architectural traits might differentially respond to the soil microenvironment and may change according to the plant needs. For this reason, roots have a relevant role in the adaptation of plants under abiotic stress conditions. The development of an efficient root system is fundamental to improve crop productivity, and studying plant genotypes regarding the root traits associated with better adaptation to specific stresses is one of the strategies being followed to achieve this aim [121]. Greater attention should be focused on enhancing crop adaptation upon diverse environmental stresses by the selection of the best adapted root system on the basis of genetic features or thanks to the “manipulation” of root system traits of crops to grow in nutrient-deficient or drought conditions ([118] and references therein). Several approaches have been developed for phenotyping root systems [122,123,124,125], although there are very few methods and technologies for high-throughput phenotyping of the root growth in the field, e.g., shovelomics [126] and RootTracker [127]. Recent technological advances in the field of phenomics allow plant breeders to successfully evaluate the root architecture of different crops, at least from younger seedlings in a controlled environment. In spite of that, the root system architecture is affected by soil and rhizosphere conditions and root morphology can also change within plants belonging to the same genotype, considering that roots show a high plasticity [125]. Additionally, biotic root traits that involve the capacity of roots to interact directly with soil biota, including AMF, should be considered as a relevant trait to be investigated. Although nowadays it is known that these microbes are characterized by a great potential for improving the sustainability and stability of agricultural productions and that a great progress has been made in understanding the AM symbiosis, considerable gaps of knowledge need to be filled in optimizing agroecosystem services associated with AMF.

#### 4.4.3. Spectranomics

Recent technological advances have led to solutions for exploring advanced methods for large-scale phenotyping data acquisition and processing [128]. Remote sensing has allowed more affordable and integrated measurements of functional traits at large scales, and natural variability in plant function and variability in response to climate change require the characterization of foliar functional traits by spectroscopy [87], 1289Particularly, spectroscopic approaches measure the spectrally detailed light reflectance and/or transmittance of plant foliage, obtaining accurate predictions of diverse functional chemical traits, such as those related to plant growth and primary metabolism as well as traits related to defense and secondary metabolism (e.g., phenolics and lignin) ([129] and references therein). Spectranomics, based on spectroscopy, allows to correlate plant canopy genotype and functional traits to their spectral-optical properties. The latter can then be combined with chemistry, taxonomy, and community ecology. The use of spectral remote sensing for biodiversity and functional ecology studies could lead to obtaining useful information for the conservation of plant communities [130]. Beneficial plant–microbe interactions might influence plant traits as spectral data that can be non-destructively identified [131].

## 5. Conclusions

**Figure 2:**
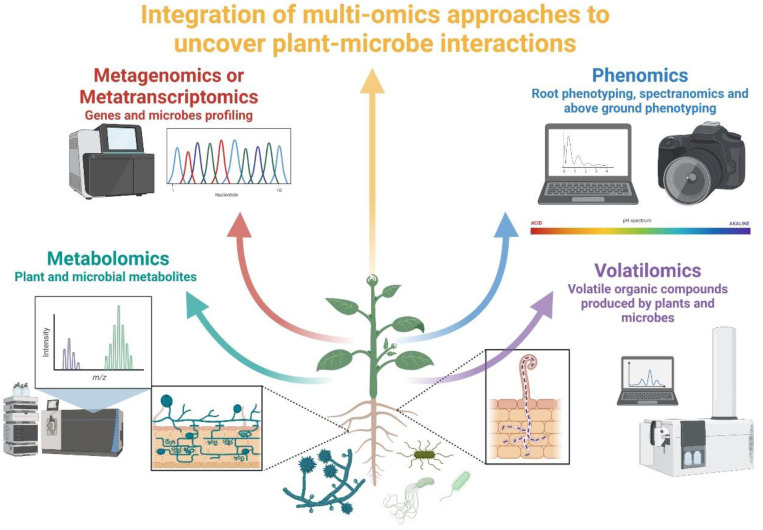
Multi-omics approaches can easily enable the understanding of complex plant–microbiome interactions. Thanks to these approaches, researchers can unveil which microbes are present and their genetic information, the root traits, the gene expression profile, metabolites, volatiles, and spectra of both plants and microbes, also during the challenge with abiotic stresses. Integrating all the information obtained from the multi-omics approaches, researchers will also better understand and exploit the staunch ally and the innovative weapon against crop abiotic stresses.

Ongoing climate change has been increasing the negative impact of abiotic stresses on plants, limiting agricultural productivity. Abiotic stress tolerance is a polygenic response where sensing, signal transduction, and expression of stress-responsive genes are involved. In this review, the belowground microbiome has been explored as a promising tool for inducing and enhancing this tolerance and, particularly, the advantage of a multi-omics approach has been underlined. Mycorrhizal fungi and Actinomycetes have been suggested as plant allies in dealing with harsh environments, and a multi-omics technique could contribute to the fundamental goal of unearthing and exploiting their beneficial interactions with plants in agrosystems. By integrating diverse omics approaches, it is possible to understand: (i) who is present and its genetic background, (ii) what changes occur in the gene expression, metabolites, volatiles, and spectra, and how roots’ architecture and plasticity is modified, in response to abiotic stresses, and (iii) how root-associated microorganisms mediate the host plant responses to a stress condition (Figure 2). The development of integrative databases to access the data produced by a multi-omics approach might lead to the identification of regulatory networks linking genome-wide transcriptomics, proteomics, and metabolomics profiles in crops exposed to abiotic stresses, including key genes (negative and positive regulators) associated with the biosynthesis of effective metabolites. A wider application of these techniques would in fact allow the identification of novel plant and microbial bioactive molecules that could be exploited in sustainable agriculture.

## Data Availability

Not applicable.

## Supplementary Materials

The following supporting information can be downloaded at: https://www.mdpi.com/article/10.3390/ijms23031091/s1.

## References

[1] Berg G., Rybakova D., Grube M., Köberl M. (2016). The plant microbiome explored: Implications for experimental botany. J. Exp. Bot..

[2] Sharma M., Sudheer S., Usmani Z., Rani R., Gupta P. (2020). Deciphering the omics of plant-microbe interaction: Perspectives and new insights. Curr. Genom..

[3] Hacquard S. (2016). Disentangling the factors shaping microbiota composition across the plant holobiont. New Phytol..

[4] Beattie G.A. (2015). Microbiomes: Curating communities from plants. Nature.

[5] Compant S., Samad A., Faist H., Sessitsch A. (2019). A review on the plant microbiome: Ecology, functions, and emerging trends in microbial application. J. Adv. Res..

[6] Campisano A., Antonielli L., Pancher M., Yousaf S., Pindo M., Pertot I. (2014). Bacterial endophytic communities in the grapevine depend on pest management. PLoS ONE.

[7] Mahajan S., Tuteja N. (2005). Cold, salinity and drought stresses: An overview. Arch. Biochem. Biophys..

[8] Chialva M., Salvioli A., Daghino S., Ghignone S., Bagnaresi P., Chiapello M., Novero M., Spadaro D., Perotto S., Bonfante P. (2018). Native soils with their microbiotas elicit a state of alert in tomato plants. New Phytol..

[9] Gouda S., Kerry R.G., Das G., Paramithiotis S., Shin H.S., Patra J.K. (2018). Revitalization of plant growth promoting rhizobacteria for sustainable development in agriculture. Microbiol. Res..

[10] Berruti A., Lumini E., Balestrini R., Bianciotto V. (2016). Arbuscular mycorrhizal fungi as natural biofertilizers: Let’s benefit from past successes. Front. Microbiol..

[11] Smith S.E., Read D.J. (2008). Mycorrhizal Symbiosis.

[12] Balestrini R., Lumini E. (2018). Focus on mycorrhizal symbioses. Appl. Soil Ecol..

[13] Spatafora J.W., Chang Y., Benny G.L., Lazarus K., Smith M.E., Berbee M.L., Bonito G., Corradi N., Grigoriev I., Gryganskyi A. (2016). A phylum-level phylogenetic classification of zygomycete fungi based on genome-scale data. Mycologia.

[14] Olanrewaju O.S., Babalola O.O. (2019). Streptomyces: Implications and interactions in plant growth promotion. Appl. Microbiol. Biotechnol..

[15] Barka E.A., Vatsa P., Sanchez L., Gaveau-Vaillant N., Jacquard C., Klenk H.P., Clément C., Ouhdouch Y., van Wezel G.P. (2016). Taxonomy, physiology, and natural products of actinobacteria. Microbiol. Mol. Biol. Rev..

[16] Bhatti A.A., Haq S., Bhat R.A. (2017). Actinomycetes benefaction role in soil and plant health. Microb. Pathog..

[17] Chukwuneme C.F., Babalola O.O., Kutu F.R., Ojuederie O.B. (2020). Characterization of actinomycetes isolates for plant growth promoting traits and their effects on drought tolerance in maize. J. Plant Interact..

[18] Nimnoi P., Pongsilp N., Lumyong S. (2010). Endophytic actinomycetes isolated from *Aquilaria crassna* Pierre ex Lec and screening of plant growth promoters production. World J. Microbiol. Biotechnol..

[19] Cramer G.R., Urano K., Delrot S., Pezzotti M., Shinozaki K. (2011). Effects of abiotic stress on plants: A systems biology perspective. BMC Plant Biol..

[20] Lohani N., Jain D., Singh M.B., Bhalla P.L. (2020). Engineering multiple abiotic Stress tolerance in canola, *Brassica napus*. Front. Plant Sci..

[21] Trenberth K.E., Dai A., Van Der Schrier G., Jones P.D., Barichivich J., Briffa K.R., Sheffield J. (2014). Global warming and changes in drought. Nat. Clim. Chang..

[22] Evelin H., Devi T.S., Gupta S., Kapoor R. (2019). Mitigation of salinity stress in plants by arbuscular mycorrhizal symbiosis: Current understanding and new challenges. Front. Plant Sci..

[23] Nephali L., Piater L.A., Dubery I.A., Patterson V., Huyser J., Burgess K., Tugizimana F. (2020). Biostimulants for plant growth and mitigation of abiotic stresses: A metabolomics perspective. Metabolites.

[24] Westman S.M., Kloth K.J., Hanson J., Ohlsson A.B., Albrectsen B.R. (2019). Defense priming in *Arabidopsis*—A Meta-Analysis. Sci. Rep..

[25] Ruelland E., Zachowski A. (2010). How plants sense temperature. Environ. Exp. Bot..

[26] Martinez-Medina A., Flors V., Heil M., Mauch-mani B., Pieterse C.M.J. (2016). Recognizing plant defense priming. Trends Microbiol..

[27] Baxter A., Mittler R., Suzuki N. (2014). ROS as key players in plant stress signalling. J. Exp. Bot..

[28] Choudhury F.K., Rivero R.M., Blumwald E., Mittler R. (2017). Reactive oxygen species, abiotic stress and stress combination. Plant J..

[29] Nahar K., Hasanuzzaman M., Fujita M., Iqbal N., Nazar R. (2015). Roles of osmolytes in plant adaptation to drought and salinity. Osmolytes and Plants Acclimation to Changing Environment: Emerging Omics Technologies.

[30] Plett J.M., Martin F.M. (2018). Know your enemy, embrace your friend: Using omics to understand how plants respond differently to pathogenic and mutualistic microorganisms. Plant J..

[31] Balestrini R., Chitarra W., Fotopoulos V., Ruocco M., Lukac M., Grenni P., Gamboni M. (2017). Potential Role of Beneficial Soil Microorganisms in Plant Tolerance to Abiotic Stress Factors.

[32] Antoine S., Hériché M., Boussageon R., Noceto P.A., van Tuinen D., Wipf D., Courty P.E. (2021). A historical perspective on mycorrhizal mutualism emphasizing arbuscular mycorrhizas and their emerging challenges. Mycorrhiza.

[33] Begum N., Qin C., Ahanger M.A., Raza S., Khan M.I., Ashraf M., Ahmed N., Zhang L. (2019). Role of arbuscular mycorrhizal fungi in plant growth regulation: Implications in abiotic stress tolerance. Front. Plant Sci..

[34] Chandrasekaran M., Boopathi T., Manivannan P. (2021). Comprehensive assessment of ameliorative effects of AMF in alleviating abiotic stress in tomato plants. J. Fungi.

[35] Kosová K., Vítámvás P., Urban M.O., Prášil I.T. (2013). Plant proteome responses to salinity stress—Comparison of glycophytes and halophytes. Funct. Plant Biol..

[36] Pan J., Peng F., Tedeschi A., Xue X., Wang T., Liao J., Zhang W., Huang C. (2020). Do halophytes and glycophytes differ in their interactions with arbuscular mycorrhizal fungi under salt stress? A meta-analysis. Bot. Stud..

[37] Augé R.M., Toler H.D., Saxton A.M. (2014). Arbuscular mycorrhizal symbiosis and osmotic adjustment in response to NaCl stress: A meta-analysis. Front. Plant Sci..

[38] Chandrasekaran M., Boughattas S., Hu S., Oh S.H., Sa T. (2014). A meta-analysis of arbuscular mycorrhizal effects on plants grown under salt stress. Mycorrhiza.

[39] Evelin H., Giri B., Kapoor R. (2013). Ultrastructural evidence for AMF mediated salt stress mitigation in *Trigonella foenum-graecum*. Mycorrhiza.

[40] Evelin H., Kapoor R., Giri B. (2009). Arbuscular mycorrhizal fungi in alleviation of salt stress: A review. Ann. Bot..

[41] Eroğlu Ç.G., Cabral C., Ravnskov S., Bak Topbjerg H., Wollenweber B. (2020). Arbuscular mycorrhiza influences carbon-use efficiency and grain yield of wheat grown under pre- and post-anthesis salinity stress. Plant Biol..

[42] Pollastri S., Savvides A., Pesando M., Lumini E., Volpe M.G., Ozudogru E.A., Faccio A., De Cunzo F., Michelozzi M., Lambardi M. (2018). Impact of two arbuscular mycorrhizal fungi on *Arundo donax* L. response to salt stress. Planta.

[43] Chitarra W., Pagliarani C., Maserti B., Lumini E., Siciliano I., Cascone P., Schubert A., Gambino G., Balestrini R., Guerrieri E. (2016). Insights on the impact of arbuscular mycorrhizal symbiosis on tomato tolerance to water stress. Plant Physiol..

[44] Volpe V., Chitarra W., Cascone P., Volpe M.G., Bartolini P., Moneti G., Pieraccini G., Di Serio C., Maserti B., Guerrieri E. (2018). The association with two different arbuscular mycorrhizal fungi differently affects water stress tolerance in tomato. Front. Plant Sci..

[45] Rivero J., Álvarez D., Flors V., Azcón-Aguilar C., Pozo M.J. (2018). Root metabolic plasticity underlies functional diversity in mycorrhiza-enhanced stress tolerance in tomato. New Phytol..

[46] Begum N., Ahanger M.A., Su Y., Lei Y., Mustafa N.S.A., Ahmad P., Zhang L. (2019). Improved drought tolerance by AMF inoculation in maize (*Zea mays*) involves physiological and biochemical implications. Plants.

[47] Balestrini R., Brunetti C., Chitarra W., Nerva L. (2020). Photosynthetic traits and nitrogen uptake in crops: Which is the role of arbuscular mycorrhizal fungi?. Plants.

[48] Mathur S., Tomar R.S., Jajoo A. (2019). Arbuscular mycorrhizal fungi (AMF) protects photosynthetic apparatus of wheat under drought stress. Photosynth. Res..

[49] Zou Y.N., Wu Q.S., Kuča K. (2021). Unravelling the role of arbuscular mycorrhizal fungi in mitigating the oxidative burst of plants under drought stress. Plant Biol..

[50] Qin Y., Druzhinina I.S., Pan X., Yuan Z. (2016). Microbially mediated plant salt tolerance and microbiome-based solutions for saline agriculture. Biotechnol. Adv..

[51] Güimil S., Chang H.S., Zhu T., Sesma A., Osbourn A., Roux C., Ioannidis V., Oakeley E.J., Docquier M., Descombes P. (2005). Comparative transcriptomics of rice reveals an ancient pattern of response to microbial colonization. Proc. Natl. Acad. Sci. USA.

[52] Fiorilli V., Vallino M., Biselli C., Faccio A., Bagnaresi P., Bonfante P. (2015). Host and non-host roots in rice: Cellular and molecular approaches reveal differential responses to arbuscular mycorrhizal fungi. Front. Plant Sci..

[53] Fiorilli V., Catoni M., Miozzi L., Novero M., Accotto G.P., Lanfranco L. (2009). Global and cell-type gene expression profiles in tomato plants colonized by an arbuscular mycorrhizal fungus. New Phytol..

[54] Balestrini R., Salvioli A., Dal Molin A., Novero M., Gabelli G., Papparelli E., Marroni F., Bonfante P. (2017). Impact of an arbuscular mycorrhizal fungus versus a mixed microbial inoculum on the transcriptome reprogramming of grapevine roots. Mycorrhiza.

[55] Fiorilli V., Vannini C., Ortolani F., Garcia-Seco D., Chiapello M., Novero M., Domingo G., Terzi V., Morcia C., Bagnaresi P. (2018). Omics approaches revealed how arbuscular mycorrhizal symbiosis enhances yield and resistance to leaf pathogen in wheat. Sci. Rep..

[56] Balestrini R., Rosso L.C., Veronico P., Melillo M.T., De Luca F., Fanelli E., Colagiero M., Salvioli A., Ciancio A., Pentimone I. (2019). Transcriptomic responses to water deficit and nematode infection in mycorrhizal tomato roots. Front. Microbiol..

[57] Grover M., Bodhankar S., Maheswari M., Srinivasarao C., Subramaniam G., Arumugam S., Rajendran V. (2016). Actinomycetes as mitigators of climate change and abiotic stress. Plant Growth Promoting Actinobacteria.

[58] Yandigeri M.S., Meena K.K., Singh D., Malviya N., Singh D.P., Solanki M.K., Yadav A.K., Arora D.K. (2012). Drought-tolerant endophytic Actinobacteria promote growth of wheat (*Triticum aestivum*) under water stress conditions. Plant Growth Regul..

[59] Aly M.M., El-Sabbagh S.M., El-Shouny W.A., Ebrahim M.K.H. (2003). Physiological response of *Zea mays* to NaCl stress with respect to *Azotobacter chroococcum* and *Streptomyces niveus*. Pak. J. Biol. Sci..

[60] Palaniyandi S.A., Damodharan K., Yang S.H., Suh J.W. (2014). *Streptomyces* sp. Strain PGPA39 alleviates salt stress and promotes growth of ‘Micro Tom’ tomato plants. J. Appl. Microbiol..

[61] Hasegawa S., Meguro A., Toyoda K., Nishimura T., Kunoh H. (2005). Drought tolerance of tissue-cultured seedlings of mountain laurel (*Kalmia latifolia* L.) induced by an endophytic actinomycete II. Acceleration of callose accumulation and lignification. Actinomycetologica.

[62] Lauber C.L., Hamady M., Knight R., Fierer N. (2009). Pyrosequencing-based assessment of soil pH as a predictor of soil bacterial community structure at the continental scale. Appl. Environ. Microbiol..

[63] Zarraonaindia I., Owens S.M., Weisenhorn P., West K., Hampton-Marcell J., Lax S., Bokulich N.A., Mills D.A., Martin G., Taghavi S. (2015). The soil microbiome influences grapevine-associated microbiota. mBio.

[64] Johnston-Monje D., Lundberg D.S., Lazarovits G., Reis V.M., Raizada M.N. (2016). Bacterial populations in juvenile maize rhizospheres originate from both seed and soil. Plant Soil.

[65] White R.A., Rivas-Ubach A., Borkum M.I., Köberl M., Bilbao A., Colby S.M., Hoyt D.W., Bingol K., Kim Y.M., Wendler J.P. (2017). The state of rhizospheric science in the era of multi-omics: A practical guide to omics technologies. Rhizosphere.

[66] Alawiye T.T., Babalola O.O. (2019). Bacterial diversity and community structure in typical plant rhizosphere. Diversity.

[67] Priya P., Aneesh B., Harikrishnan K. (2021). Genomics as a potential tool to unravel the rhizosphere microbiome interactions on plant health. J. Microbiol. Methods.

[68] Anderson J.T., Song B. (2020). Plant adaptation to climate change—Where are we?. J. Syst. Evol..

[69] Palit P., Kudapa H., Zougmore R., Kholova J., Whitbread A., Sharma M., Varshney R.K. (2020). An integrated research framework combining genomics, systems biology, physiology, modelling and breeding for legume improvement in response to elevated CO_2_ under climate change scenario. Curr. Plant Biol..

[70] Li Q., Yan J. (2020). Sustainable agriculture in the era of omics: Knowledge-driven crop breeding. Genome Biol..

[71] Levy A., Conway J.M., Dangl J.L., Woyke T. (2018). Elucidating bacterial gene functions in the plant microbiome. Cell Host Microbe.

[72] Khatabi B., Gharechahi J., Ghaffari M.R., Liu D., Haynes P.A., McKay M.J., Mirzaei M., Salekdeh G.H. (2019). Plant-microbe symbiosis: What has proteomics taught us?. Proteomics.

[73] Chaston J., Douglas A.E. (2012). Making the most of “omics” for symbiosis research. Biol. Bull..

[74] Berg G., Rybakova D., Fischer D., Cernava T., Vergès M.C., Charles T., Chen X., Cocolin L., Eversole K., Corral G.H. (2020). Microbiome definition re-visited: Old concepts and new challenges. Microbiome.

[75] Nilsson R.H., Anslan S., Bahram M., Wurzbacher C., Baldrian P., Tedersoo L. (2019). Mycobiome diversity: High-throughput sequencing and identification of fungi. Nat. Rev. Microbiol..

[76] Escobar-Zepeda A., Vera-Ponce de León A., Sanchez-Flores A. (2015). The road to metagenomics: From microbiology to DNA sequencing technologies and bioinformatics. Front. Genet..

[77] Singer E., Bushnell B., Coleman-Derr D., Bowman B., Bowers R.M., Levy A., Gies E.A., Cheng J.F., Copeland A., Klenk H.P. (2016). High-resolution phylogenetic microbial community profiling. ISME J..

[78] Vogel T.M., Hirsch P.R., Simonet P., Jansson J.K., Tiedje J.M., van Elsas J.D., Nalin R., Philippot L., Bailey M.J. (2009). Advantages of the metagenomic approach for soil exploration: Reply from Vogel et al. Nat. Rev. Microbiol..

[79] Solden L., Lloyd K., Wrighton K. (2016). The bright side of microbial dark matter: Lessons learned from the uncultivated majority. Curr. Opin. Microbiol..

[80] Quince C., Walker A.W., Simpson J.T., Loman N.J., Segata N. (2017). Shotgun metagenomics, from sampling to analysis. Nat. Biotechnol..

[81] Xu J. (2016). Fungal DNA barcoding. Genome.

[82] Öpik M., Metsis M., Daniell T.J., Zobel M., Moora M. (2009). Large-scale parallel 454 sequencing reveals host ecological group specificity of arbuscular mycorrhizal fungi in a boreonemoral forest. New Phytol..

[83] Lumini E., Orgiazzi A., Borriello R., Bonfante P., Bianciotto V. (2010). Disclosing arbuscular mycorrhizal fungal biodiversity in soil through a land-use gradient using a pyrosequencing approach. Environ. Microbiol..

[84] Victorino Í.M.M., Berruti A., Orgiazzi A., Voyron S., Bianciotto V., Lumini E., Ferrol N., Lanfranco L. (2020). High-throughput DNA sequence-based analysis of AMF communities. Arbuscular Mycorrhizal Fungi. Methods in Molecular Biology.

[85] Zepeda Mendoza M.L., Sicheritz-Pontén T., Gilbert M.T. (2015). Environmental genes and genomes: Understanding the differences and challenges in the approaches and software for their analyses. Brief. Bioinform..

[86] Tedersoo L., Anslan S., Bahram M., Põlme S., Riit T., Liiv I., Kõljalg U., Kisand V., Nilsson R.H., Hildebrand F. (2015). Shotgun metagenomes and multiple primer pair-barcode combinations of amplicons reveal biases in metabarcoding analyses of fungi. MycoKeys.

[87] Crandall S.G., Gold K.M., Jiménez-Gasco M.D.M., Filgueiras C.C., Willett D.S. (2020). A multi-omics approach to solving problems in plant disease ecology. PLoS ONE.

[88] Kuske C.R., Hesse C.N., Challacombe J.F., Cullen D., Herr J.R., Mueller R.C., Tsang A., Vilgalys R. (2015). Prospects and challenges for fungal metatranscriptomics of complex communities. Fungal Ecol..

[89] Bastida F., Hernández T., García C. (2014). Metaproteomics of soils from semiarid environment: Functional and phylogenetic information obtained with different protein extraction methods. J. Proteom..

[90] Wilmes P., Bond P.L. (2004). The application of twodimensional polyacrylamide gel electrophoresis and downstream analyses to a mixed community of prokaryotic microorganisms. Environ. Microbiol..

[91] Marcelino V.R., Irinyi L., Eden J.S., Meyer W., Holmes E.C., Sorrell T.C. (2019). Metatranscriptomics as a tool to identify fungal species and subspecies in mixed communities—A proof of concept under laboratory conditions. IMA Fungus.

[92] Chialva M., Ghignone S., Novero M., Hozzein W.N., Lanfranco L., Bonfante P. (2019). Tomato RNA-seq data mining reveals the taxonomic and functional diversity of root-associated microbiota. Microorganisms.

[93] Chiapello M., Zampieri E., Mello A. (2020). A small effort for researchers, a big gain for soil metaproteomics. Front. Microbiol..

[94] Martinez-Alonso E., Pena-Perez S., Serrano S., Garcia-Lopez E., Alcazar A., Cid C. (2019). Taxonomic and functional characterization of a microbial community from a volcanic englacial ecosystem in Deception Island, Antarctica. Sci. Rep..

[95] Zampieri E., Chiapello M., Daghino S., Bonfante P., Mello A. (2016). Soil metaproteomics reveals an inter-kingdom stress response to the presence of black truffles. Sci. Rep..

[96] Nilsson R.H., Larsson K.H., Taylor A.F.S., Bengtsson-Palme J., Jeppesen T.S., Schigel D., Kennedy P., Picard K., Glöckner F.O., Tedersoo L. (2019). The UNITE database for molecular identification of fungi: Handling dark taxa and parallel taxonomic classifications. Nucleic Acids Res..

[97] Perez-Riverol Y., Csordas A., Bai J., Bernal-Llinares M., Hewapathirana S., Kundu D.J., Inuganti A., Jarnuczak A.F., Ternent T., Brazma A. (2019). The PRIDE database and related tools and resources in 2019: Improving support for quantification data. Nucleic Acids Res..

[98] Liu X., Locasale J.W. (2017). Metabolomics: A primer. Trends Biochem. Sci..

[99] Roberts L.D., Souza A.L., Gerszten R.E., Clish C.B. (2012). Targeted metabolomics. Curr. Protoc. Mol. Biol..

[100] Schrimpe-Rutledge A.C., Codreanu S.G., Sherrod S.D., McLean J.A. (2016). Untargeted metabolomics strategies—Challenges and emerging directions. J. Am. Soc. Mass. Spectrom..

[101] Rivero J., Gamir J., Pozo M.J., Flors V. (2015). Metabolic transition in mycorrhizal tomato roots. Front. Microbiol..

[102] Tugizimana F., Mhlongo M.I., Piater L.A., Dubery I.A. (2018). Metabolomics in plant priming research: The way forward?. Int. J. Mol. Sci..

[103] Bernardo L., Carletti P., Badeck F.W., Rizza F., Morcia C., Ghizzoni R., Rouphael Y., Colla G., Terzi V., Lucini L. (2019). Metabolomic responses triggered by arbuscular mycorrhiza enhance tolerance to water stress in wheat cultivars. Plant Physiol. Biochem..

[104] Rivero J., Lidoy J., Llopis-Giménex A., Herrero S., Flors V., Pozo M.J. (2021). Mycorrhizal symbiosis primes the accumulation of antiherbivore compounds and enhances herbivore mortality in tomato. J. Exp. Bot..

[105] Escudero-Martinez C., Bulgarelli D. (2019). Tracing the evolutionary routes of plant-microbiota interactions. Curr. Opin. Microbiol..

[106] Iannucci A., Fragasso M., Beleggia R., Nigro F., Papa R. (2017). Evolution of the crop rhizosphere: Impact of domestication on root exudates in tetraploid wheat (*Triticum turgidum* L.). Front. Plant Sci..

[107] Fiorani F., Schurr U. (2013). Future scenarios for plant phenotyping. Annu. Rev. Plant Biol..

[108] Tardieu F., Cabrera-Bosquet L., Pridmore T., Bennett M. (2017). Plant phenomics, from sensors to knowledge. Curr. Biol..

[109] Costa C., Schurr U., Loreto F., Menesatti P., Carpentier S. (2019). Plant phenotyping research trends, a science mapping approach. Front. Plant Sci..

[110] Costa J.M., Marques da Silva J., Pinheiro C., Barón M., Mylona P., Centritto M., Haworth M., Loreto F., Uzilday B., Turkan I. (2019). Opportunities and limitations of crop phenotyping in Southern European countries. Front. Plant Sci..

[111] Mhlongo M.I., Piater L.A., Madala N.E., Labuschagne N., Dubery I.A. (2018). The chemistry of plant–microbe interactions in the rhizosphere and the potential for metabolomics to reveal signaling related to defense priming and induced systemic resistance. Front. Plant Sci..

[112] Catola S., Centritto M., Cascone P., Ranieri A., Loreto F., Calamai L., Balestrini R., Guerrieri E. (2018). Effects of single or combined water deficit and aphid attack on tomato volatile organic compound (VOC) emission and plant-plant communication. Environ. Exp. Bot..

[113] Brilli F., Loreto F., Baccelli I. (2019). Exploiting plant volatile organic compounds (VOCs) in agriculture to improve sustainable defense strategies and productivity of crops. Front. Plant Sci..

[114] Bailly A., Weisskopf L. (2017). Mining the volatilomes of plant-associated microbiota for new biocontrol solutions. Front. Microbiol..

[115] Fincheira P., Quiroz A., Tortella G., Diez M.C., Rubilar O. (2021). Current advances in plant-microbe communication via volatile organic compounds as an innovative strategy to improve plant growth. Microb. Res..

[116] Kanchiswamy C.N., Malnoy M., Maffei M.E. (2015). Chemical diversity of microbial volatiles and their potential for plant growth and productivity. Front. Plant Sci..

[117] Romano A., Hanna G.B. (2018). Identification and quantification of VOCs by proton transfer reaction time of flight mass spectrometry: An experimental workflow for the optimization of specificity, sensitivity, and accuracy. J. Mass Spectrom..

[118] Arifuzzaman M., Oladzadabbasabadi A., McClean P., Rahman M. (2019). Shovelomics for phenotyping root architectural traits of rapeseed/canola (*Brassica napus* L.) and genome-wide association mapping. Mol. Genet. Genom..

[119] Lynch J.P. (2007). Roots of the second green revolution. Aust. J. Bot..

[120] Kuijken R.C., van Eeuwijk F.A., Marcelis L.F., Bouwmeester H.J. (2015). Root phenotyping: From component trait in the lab to breeding. J. Exp. Bot..

[121] Lynch J.P. (2013). Steep, cheap and deep: An ideotype to optimize water and N acquisition by maize root systems. Ann. Bot..

[122] Atkinson J.A., Pound M.P., Bennett M.J., Wells D.M. (2019). Uncovering the hidden half of plants using new advances in root phenotyping. Curr. Opin. Biotechnol..

[123] Guimarães P.H.R., de Lima I.P., de Castro A.P., Lanna A.C., Guimarães Santos Melo P., de Raïssac M. (2020). Phenotyping root systems in a set of Japonica rice accessions: Can structural traits predict the response to drought?. Rice.

[124] McGrail R.K., Van Sanford D.A., McNear D.H. (2020). Trait-based root phenotyping as a necessary tool for crop selection and improvement. Agronomy.

[125] Takahashi H., Pradal C. (2021). Root phenotyping: Important and minimum information required for root modeling in crop plants. Breed. Sci..

[126] Trachsel S., Kaeppler S.M., Brown K.M., Lynch J.P. (2011). Shovelomics: High throughput phenotyping of maize (*Zea mays* L.) root architecture in the field. Plant Soil.

[127] Aguilar J.J., Moore M., Johnson L., Greenhut R.F., Rogers E., Walker D., O’Neil F., Edwards J.L., Thystrup J., Farrow S. (2021). Capturing in-field root system dynamics with RootTracker. Plant Physiol..

[128] Yang W., Feng H., Zhang X., Zhang J., Doonan J.H., Batchelor W.D., Xiong L., Yan J. (2020). Crop phenomics and high-throughput phenotyping: Past decades, current challenges, and future perspectives. Mol. Plant.

[129] Fine P.V.A., Salazar D., Martin R.E., Metz M.R., Misiewicz T.M., Asner G.P. (2021). Exploring the links between secondary metabolites and leaf spectral reflectance in a diverse genus of Amazonian trees. Ecosphere.

[130] Asner G.P., Martin R.E. (2016). Spectranomics: Emerging science and conservation opportunities at the interface of biodiversity and remote sensing. Glob. Ecol. Conserv..

[131] Fisher J.B., Sweeney S., Brzostek E.R., Evans T.P., Johnson D.J., Myers J.A., Bourg N.A., Wolf A.T., Howe R.W., Phillips R.P. (2016). Tree-mycorrhizal associations detected remotely from canopy spectral properties. Glob. Chang. Biol..

