# Supplementary material for: Abiotic Stress and Belowground Microbiome: The Potential of Omics Approaches
Source: Int J Mol Sci. 2022 Jan 19;23(3):1091. doi: 10.3390/ijms23031091 (PMC8835006; doi:10.3390/ijms23031091)
Supplement: Supplementary file 1 [file ijms-23-01091-s001.zip › ijms-1537790-supplementary.pdf]

International Journal of
*Molecular Sciences*

MDPI

*Review*

# Abiotic stress and belowground microbiome: the potential of -omics approaches

**Marco Sandrini[1], Luca Nerva[1,2], Fabiano Sillo[2], Raffaella Balestrini[2*], Walter Chitarra[1,2] and Elisa Zampieri[2]**

**Table S1.** List of manuscripts that combine the presence of plant, AMF or Actinobacteria and -omics, *i.e.*, RNAseq or untargeted metabolomics/proteomics, in the last ten years.

| Reference | Plant | AMF and Actino-bacteria | Tissue | -Omics | Stress |
|---|---|---|---|---|---|
| **AMF** | | | | | |
| [132] | Tomato wild-type and rmc mutant | *Glomus intraradices* (former name) | Roots | Transcriptomics RNAseq | |
| [133] | Tomato (*Solanum lycopersicum*) | *Funneliformis mosseae* | Fruits | Transcriptomics RNA-Seq | |
| [52] | Rice (*Oryza sativa*) | *Rhizophagus irregularis* | Large lateral and fine lateral roots | Transcriptomics RNA-seq | |
| [134] | *Lotus japonicus* | *Rhizophagus irregularis* | Roots | Transcriptomics RNA-Seq | |
| [135] | Litchi (*Litchi chinensis*) | *Rhizophagus irregularis* | Roots | Transcriptomics RNA-seq | |
| [54] | Grapevine (*Vitis vinifera*) | *Funneliformis mossea* or mixed commercial inoculum | Roots | Transcriptomics RNA-seq | |
| [136] | Trifoliate orange (*Poncirus trifoliate*) | *Rhizophagus irregularis* | Lateral roots | Transcriptomics RNA-Seq | |
| [137] | *Lotus japonicus* | *Rhizophagus irregularis* | Roots | Transcriptomics RNA-Seq | |
| [55] | Wheat (*Triticum aestivum*) | *Funneliformis mosseae* | Roots and leaves | Transcriptomics RNA-seq Proteomics LC-MS/MS | *Xanthomonas translucens* infection |
| [138] | Cucumber (*Cucumis sativus*) | *Rhizophagus irregularis* | Roots | Transcriptomics RNA-seq | Cold stress |
| [139] | Common bean *(Phaseolus vulgaris)* | A mix of *Glomus clarum, Acaulospora scrobiculata, Gigaspora rosea* | Roots/ validation on LMD samples | Transcriptomics RNA-Seq | Water deficit |
| [140] | Sunflower *(Helianthus annuus)* | *Rhizoglomus irregulare* | Roots | Transcriptomics RNA-Seq | |

| | | | | | |
|---|---|---|---|---|---|
| [141] | *Medicago truncatula, Nicotiana benthamiana* and *Allium schoenoprasum* | *Rhizophagus irregularis* | Roots | Transcriptomics RNA-Seq combined with LMD | |
| [56] | Tomato (*Solanum lycopersicum*) | *Rhizophagus intraradices* | Roots | Transcriptomics RNA-seq | Water stress *Meloidogyne incognita* colonization |
| [142] | Maize (*Zea mays*) | *Glomus intraradices* (former name) | Roots | Transcriptomics RNA-seq | Cadmium |
| [143] | Maize (*Zea mays*) | *Rhizophagus irregularis* | Roots | Transcriptomics RNA-Seq | |
| [144] | Alfalfa (*Medicago sativa*) | *Rhizophagus intraradices* | Leaves | Transcriptomics RNA-Seq | *Acyrthosiphon pisum* attack |
| [145] | Cassava *(Manihot esculenta)* | *Rhizophagus irregularis* | Fine roots | Transcriptomics RNA-Seq | |
| [146] | *Sesbania cannabina* | *Funneliformis mosseae* | Roots/shoots | Transcriptomics RNA-Seq | Salinity |
| [147] | Sunflower *(Helianthus annuus)* | *Rhizoglomus irregulare* | Roots | Transcriptomics RNA-Seq | |
| [148] | Asparagus (*Asparagus officinalis)* | *Rhizophagus irregularis* | Leaves | Transcriptomics RNA-Seq | Salinity |
| [149] | garden pea (*Pisum sativum*) | *Rhizophagus irregularis* | Roots | Transcriptomics RNA-Seq | |
| [150] | Soybean (*Glycine max*) | *Funneliformis mosseae* | Roots | Transcriptomics RNA-Seq Metabolomics UPLC-MS/MS | Root rot disease |
| [151] | Wheat (*Triticum aestivum*) | *Funneliformis mosseae* | Roots | Transcriptomics RNA-seq | Water deficit |
| [152] | Tomato (*Solanum lycopersicum*) | *Rhizophagus irregularis* | Mature, green, red fruits | Transcriptomics RNA-Seq | |
| [153] | Sunflower *(Helianthus annuus)* | *Rhizoglomus irregulare* | Roots | Transcriptomics RNA-Seq | |
| [154] | Soybean (*Glycine max*) | *Funneliformis mosseae* | Roots | Transcriptomics RNA-Seq Proteomics iTRAQ | |
| [155] | Pea (*Pisum sativum*) | *Rhizophagus irregularis* | Roots | Transcriptomics RNA-Seq | |

| | | | | | |
|---|---|---|---|---|---|
| [156] | Alfalfa (*Medicago sativa*) | *Rhizophagus intraradices* | Leaves | Transcriptomics RNA-Seq | *Phoma medicaginis* infection |
| [157] | Tomato (*Solanum lycopersicum*) | *Rhizophagus irregularis* | Roots | Transcriptomics RNA-Seq | |
| [158] | *Medicago truncatula* | *Glomus intraradices* (former name) | LMD cell-type populations from roots | Proteomics LC-MS/MS | |
| [159] | Sorghum (*Sorghum bicolor*) | A mix of *Glomus aggregatum* and *Glomus etunicatum, Funelliformis mosseae, Rhizophagus irregularis* alone or plus PGPB | Roots | Proteomics LC-MS/MS | |
| [160] | *Medicago sativa* | *Funneliformis mosseae* | Roots | Proteomics iTRAQ HPLC | Atrazine |
| [161] | Soybean (*Glycine max*) | *Funneliformis mosseae* | Roots | Proteomics iTRAQ and LC-MS/MS | Root rot disease |
| [162] | *Echinacea angustifolia* | *Rhizophagus irregularis* | Leaves | Proteomics LC-MS/MS | Salinity |
| [163] | Pea (*Pisum sativum* | *Rhizophagus irregularis* | Seeds | Proteomics LC-MS | |
| [164] | Pea (*Pisum sativum*) | A mix of *Claroideoglomus etunicatum, Claroideoglomus claroideum, Rhizophagus irregularis, Funneliformis geosporus, Funneliformis mosseae* | Seeds | Proteomics Nano ESI LC-MS/MS Metabolomics MS/MS | *Didymella pinodes* infection |
| [165] | *Phragmites australis* | *Rhizophagus irregularis* | Root tips | Proteomics iTRAQ | Copper |
| [166] | *Pteris cretica var. nervosa* | *Funneliformis mosseae* | Fronds | Proteomics LC-MS/MS | Antimony |
| [167] | *Medicago truncatula* | *Rhizophagus irregularis* | LMD cell-type populations from roots | Metabolomics GC-EI/TOF-MS | |
| [45] | Tomato (*Solanum lycopersicum*) | A mix of *Funneliformis mosseae, Rhizoglomus irregulare* and *Claroideoglomus etunicatum* | Roots | Metabolomics LC ESI-MS | Drought and salinity |
| [102] | Wheat (*Triticum durum/T. aestivum*) | *Funneliformis mosseae* | Roots | Metabolomics UHPLC-ESI/QTOF-MS | Water deficit |
| [168] | Soybean (*Glycine max*) | A mix of *Funneliformis mosseae, Paraglomus* | Roots | Metabolomics GC-MS | |

| | | | | | |
|---|---|---|---|---|---|
| | | *occultum, Diversispora spurca, Glomus* sp., *Acaulospora scrobiculata, Gigaspora* sp. | | | |
| [169] | Sweet pepper (*Capsicum annuum*) | *Funneliformis mosseae, Rhizoglomus irregularis* | Leaves | Metabolomics UHPLC/Q-TOF | |
| [170] | Maize (*Zea mays*) | A mix of *Rhizoglomus irregulare, Funneliformis mosseae* plus *Trichoderma* | Leaves and roots | Metabolomics UHPLC/Q-TOF | |
| [171] | Tomato (*Solanum lycopersicum*) | A mix of *Funneliformis mosseae, Rhizophagus intraradices* plus *Trichoderma and PGPR* | Stems with young and mature leaves, and fine feeding roots | Metabolomics LC-MS | *Fusarium* crown and root rot diseases |
| [172] | Trifoliate orange (*Poncirus trifoliate*) | *Rhizophagus intraradices* | Roots | Metabolomics LC-MS/MS | Water deficit |
| | | ***Actinobacteria*** | | | |
| [173] | *Pedunculate oak (Quercus robur)* | *Streptomyces* sp. strain (AcH 505) | Leaves | Transcriptomics RNA-seq | Oak powdery mildew infection |
| [174] | *Arabidopsis thaliana* | *Arthrobacter endophyticus; Nocardiopsis alba* | Roots | Transcriptomics RNA-seq | Salinity |
| [175] | Wheat (*Triticum aestivum*) | *Arthrobacter nitroguajacolicus* | Roots | Transcriptomics RNA-seq | Salinity |
| [176] | Tomato (*Solanum lycopersicum*) | Streptomyces sp. KLBMP5084 | Leaves | Transcriptomic RNA-seq | Salinity |
| [177] | *Arabidopsis thaliana* | *Streptomyces* isolate (KB001) | Whole seedlings | Transcriptomics RNA-seq | *Sclerotinia sclerotiorum* and *Rhizoctonia solani* infection |
| [178] | Rice (*Oryza sativa*) | *Streptomyces hygroscopicus* OsiSh-2 | Leaves | Proteomics HPLC LC-MS/MS | *Magnaporthe oryzae* infection |
| [179] | Oats (*Avena barbata*) | Rhizosphere microbial community (*Actinomycetes*) | Roots | Metabolomics UHPLC; LC-MS/MS; LC-MS | |

| | | | | | |
|---|---|---|---|---|---|
| [180] | Peppermint (*Mentha piperita*) | *Streptomyces*, C-2012 (*Streptomyces rimosus*) *Streptomyces*, C-801 (*Streptomyces monomycini*) | Shoots | Metabolomics GC/MS | Water stress |

**Table S2:** List of manuscripts that combine the presence of AMF or Actinobacteria and -omics, *i.e.*, genome sequencing, RNAseq or untargeted metabolomics/proteomics, in the last ten years.

| Reference | AMF and Actinobacteria | -Omics | Stress |
|---|---|---|---|
| **AMF** | | | |
| [181] | *Rhizophagus irregularis* DAOM197198 | Genome sequencing | |
| [182] | *Rhizophagus irregularis* DAOM197198 | Single nucleus genome sequencing | |
| [183] | *Gigaspora rosea* | Genome sequencing | |
| [184] | *Acaulospora morrowiae, Diversispora versiforme, Scutellospora calospora, Racocetra castanea, Paraglomus brasilianum, Ambispora leptoticha, Claroideoglomus claroideum, Funneliformis mosseae* | Transcriptomics Ultra-low input RNA-seq | |
| [185] | *Rhizophagus irregularis* DAOM197198 | Genome assembly and gene annotation, and compared its gene content with five isolates of *R. irregularis* sampled in the same field. | |
| [186] | *Diversispora epigaea* (formerly *Glomus versiforme*) and its bacterial endosymbionts | Genome assembly and comparative genomics | |
| [187] | *Gigaspora rosea* | Comparative genomics | |
| [188] | *Gigaspora margarita* | Genome sequencing | |
| ***Actinobacteria*** | | | |
| [189] | *Planobispora rosea* ATCC 53733 | Transcriptomics RNA-seq Proteomics nano LC-ESI-LIT-MS/MS | |
| [190] | *Nocardiopsis gilva* YIM 90087$^{T}$ | Transcriptomics RNA-seq | Salinity |
| [191] | *Saccharothrix yanglingensis* Hhs.015 | Transcriptomics RNA-seq | *Valsa mali* |

| | | | |
|---|---|---|---|
| [192] | *Rhodococcus* sp. CNS16 | Transcriptomics RNA-seq | Temperature gradients |
| [193] | *Nocardiopsis xinjiangensis* | Proteomics iTRAQ, LC-MS/MS | Salinity |
| [194] | *Streptomyces* spp. | Metabolomics UHPLC Q-TOF | *Xanthomonas oryzae* pv. *Oryzae* infection |
| [195] | *Streptomyces fradiae* MM456M-mF7 | Metabolomics HPLC, LC-ESI-MS/MS | |
| [196] | *Thermobifida cellulosilytica* | Metabolomics LC-MS/MS | |
| [197] | *Streptomyces pulveraceus* Strain ES16 | Metabolomics HPLC-HRMS Volatilomics SPME-GC-MS | |

**References**

132)Ruzicka, D.; Chamala, S.; Barrios-Masias, F.H.; Martin, F.; Smith, S.; Jackson, L.E.; Barbazuk, W.B.; Schachtman, D.P. Inside arbuscular mycorrhizal roots - molecular probes to understand the symbiosis. *Plant Genome* **2013**, *6*, 1-13. https://doi.org/10.3835/plantgenome2012.06.0007.

133)Zouari, I.; Salvioli, A.; Chialva, M.; Novero, M.; Miozzi, L.; Tenore, G.C.; Bagnaresi, P.; Bonfante, P. From root to fruit: RNA-Seq analysis shows that arbuscular mycorrhizal symbiosis may affect tomato fruit metabolism. *BMC Genomics* **2014**, *15*, 221. https://doi.org/10.1186/1471-2164-15-221.

134)Handa, Y.; Nishide, H.; Takeda, N.; Suzuki, Y.; Kawaguchi, M.; Saito, K. RNA-seq Transcriptional Profiling of an Arbuscular Mycorrhiza Provides Insights into Regulated and Coordinated Gene Expression in *Lotus japonicus* and *Rhizophagus irregularis*. *Plant Cell Physiol* **2015**, *56*, 1490-1511. https://doi.org/10.1093/pcp/pcv071.

135)Shu, B.; Li, W.; Liu, L.; Wei, Y.; Shi, S. Transcriptomes of Arbuscular Mycorrhizal Fungi and Litchi Host Interaction after Tree Girdling. *Front Microbiol* **2016**, *7*, 408. https://doi.org/10.3389/fmicb.2016.00408.

136)Chen, W.; Li, J.; Zhu, H.; Xu, P.; Chen, J.; Yao, Q. Arbuscular Mycorrhizal Fungus Enhances Lateral Root Formation in *Poncirus trifoliata* (L.) as Revealed by RNA-Seq Analysis. *Front Plant Sci* **2017**, *8*, 2039. https://doi.org/10.3389/fpls.2017.02039.

137)Sugimura, Y.; Saito, K. Transcriptional profiling of arbuscular mycorrhizal roots exposed to high levels of phosphate reveals the repression of cell cycle-related genes and secreted protein genes in *Rhizophagus irregularis*. *Mycorrhiza* **2017**, *27*, 139-146. https://doi.org/10.1007/s00572-016-0735-y.

138)Ma, J.; Sun, C.; Bai, L.; Dong, R.; Yan, Y.; Yu, X.; He, C.; Zou, Z.; Li, Y. Transcriptome Analysis of Cucumber Roots Reveals Key Cold-Resistance Genes Induced by AM Fungi. *Plant Mol Biol Rep* **2018**, *36*, 135-148. https://doi.org/10.1007/s11105-018-1066-2.

139)Recchia, G.H.; Konzen, E.R.; Cassieri, F.; Caldas, D.G.G., Tsai, S.M. Arbuscular Mycorrhizal Symbiosis Leads to Differential Regulation of Drought-Responsive Genes in Tissue-Specific Root Cells of Common Bean. *Front Microbiol* **2018**, *9*, 1339. https://doi.org/10.3389/fmicb.2018.01339.

140)Vangelisti, A.; Natali, L.; Bernardi, R.; Sbrana, C.; Turrini, A.; Hassani-Pak, K.; Hughes, D.; Cavallini, A.; Giovannetti, M.; Giordani, T. Transcriptome changes induced by arbuscular mycorrhizal fungi in sunflower (*Helianthus annuus* L.) roots. *Sci Rep* **2018**, *8*, 4. https://doi.org/10.1038/s41598-017-18445-0.

141)Zeng, T.; Holmer, R.; Hontelez, J.; Te Lintel-Hekkert, B.; Marufu, L.; de Zeeuw, T.; Wu, F.; Schijlen, E.; Bisseling, T.; Limpens, E. Host- and stage-dependent secretome of the arbuscular mycorrhizal fungus *Rhizophagus irregularis*. *Plant J* **2018**, *94*, 411-425. https://doi.org/10.1111/tpj.13908.

142)Gu, L.; Zhao, M.; Ge, M.; Zhu, S.; Cheng, B.; Li, X. Transcriptome analysis reveals comprehensive responses to cadmium stress in maize inoculated with arbuscular mycorrhizal fungi. *Ecotoxicol Environ Saf* **2019** *186*, 109744. https://doi.org/10.1016/j.ecoenv.2019.109744.

143)Han, G.; Cheng, C.; Zheng, Y.; Wang, X.; Xu, Y.; Wang, W.; Zhu, S.; Cheng, B. Identification of Long Non-Coding RNAs and the Regulatory Network Responsive to Arbuscular Mycorrhizal Fungi Colonization in Maize Roots. *Int J Mol Sci* **2019**, *20*, 4491. https://doi.org/10.3390/ijms20184491.

144)Li, Y.; Nan, Z.; Duan, T. *Rhizophagus intraradices* promotes alfalfa (*Medicago sativa*) defense against pea aphids (*Acyrthosiphon pisum*) revealed by RNA-Seq analysis. *Mycorrhiza* **2019**, *29*, 623-635. https://doi.org/10.1007/s00572-019-00915-0.

145)Mateus, I.D.; Masclaux, F.G.; Aletti, C.; Rojas, E.C.; Savary, R.; Dupuis, C.; Sanders, I.R. Dual RNA-seq reveals large-scale non-conserved genotype × genotype-specific genetic reprograming and molecular crosstalk in the mycorrhizal symbiosis. *ISME J* **2019**, *13*, 1226-1238. https://doi.org/10.1038/s41396-018-0342-3.

146)Ren, C.G.; Kong, C.C.; Yan, K.; Xie, Z.H. Transcriptome analysis reveals the impact of arbuscular mycorrhizal symbiosis on *Sesbania cannabina* expose to high salinity. *Sci Rep* **2019**, *9*, 2780. https://doi.org/10.1038/s41598-019-39463-0.

147)Vangelisti, A.; Mascagni, F.; Giordani, T.; Sbrana, C.; Turrini, A.; Cavallini, A.; Giovannetti, M.; Natali, L. Arbuscular mycorrhizal fungi induce the expression of specific retrotransposons in roots of sunflower (*Helianthus annuus* L.). *PLoS One* **2019**, *14*, e0212371. https://doi.org/10.1371/journal.pone.0212371.

148)Zhang, X.; Han, C.; Gao, H.; Cao, Y. Comparative transcriptome analysis of the garden asparagus (*Asparagus officinalis* L.) reveals the molecular mechanism for growth with arbuscular mycorrhizal fungi under salinity stress. *Plant Physiol Biochem* **2019**, *141*, 20-29. https://doi.org/10.1016/j.plaphy.2019.05.013.

149)Afonin, A.M.; Leppyanen, I.V.; Kulaeva, O.A.; Shtark, O.Y.; Tikhonovich, I.A.; Dolgikh, E.A.; Zhukov, V.A. A high coverage reference transcriptome assembly of pea (*Pisum sativum* L.) mycorrhizal roots. *Vavilovskii Zhurnal Genet Selektsii* **2020**, *24*, 331-339. https://doi.org/10.18699/VJ20.625.

150)Lu, C.C.; Guo, N.; Yang, C.; Sun, H.B.; Cai, B.Y. Transcriptome and metabolite profiling reveals the effects of *Funneliformis mosseae* on the roots of continuously cropped soybeans. *BMC Plant Biol* **2020**, *20*, 479. https://doi.org/10.1186/s12870-020-02647-2.

151)Moradi Tarnabi, Z.; Iranbakhsh, A.; Mehregan, I.; Ahmadvand, R. Impact of arbuscular mycorrhizal fungi (AMF) on gene expression of some cell wall and membrane elements of wheat (*Triticum*

*aestivum* L.) under water deficit using transcriptome analysis. *Physiol Mol Biol Plants* **2020**, *26*, 143-162. https://doi.org/10.1007/s12298-019-00727-8.
152) Schubert, R.; Werner, S.; Cirka, H.; Rödel, P.; Tandron Moya, Y.; Mock, H.P.; Hutter, I.; Kunze, G.; Hause, B. Effects of Arbuscular Mycorrhization on Fruit Quality in Industrialized Tomato Production. *Int J Mol Sci* **2020**, *21*, 7029. https://doi.org/ 10.3390/ijms21197029.
153) Vangelisti, A.; Turrini, A.; Sbrana, C.; Avio, L.; Giordani, T.; Natali, L.; Giovannetti, M.; Cavallini, A. Gene expression in *Rhizoglomus irregulare* at two different time points of mycorrhiza establishment in *Helianthus annuus* roots, as revealed by RNA-seq analysis. *Mycorrhiza* **2020**, *30*, 373-387. https://doi.org/10.1007/s00572-020-00950-2.
154) Zhang, X.Q.; Bai, L.; Sun, H.B.; Yang, C.; Cai, B.Y. Transcriptomic and Proteomic Analysis Revealed the Effect of *Funneliformis mosseae* in Soybean Roots Differential Expression Genes and Proteins. *J Proteome Res* **2020**, *19*, 3631-3643. https://doi.org/10.1021/acs.jproteome.0c00017.
155) Zorin, E.A.; Afonin, A.M.; Kulaeva, O.A.; Gribchenko, E.S.; Shtark, O.Y.; Zhukov, V.A. Transcriptome Analysis of Alternative Splicing Events Induced by Arbuscular Mycorrhizal Fungi (*Rhizophagus irregularis*) in Pea (*Pisum sativum* L.) Roots. *Plants (Basel)* **2020**, *9*, 1700. https://doi.org/10.3390/plants9121700.
156) Li, Y.; Duan, T.; Nan, Z.; Li, Y. Arbuscular mycorrhizal fungus alleviates alfalfa leaf spots caused by *Phoma medicaginis* revealed by RNA-seq analysis. *J Appl Microbiol* **2021**, *130*, 547-560. https://doi.org/10.1111/jam.14387.
157) Vasan, S.; Srivastava, D.; Cahill, D.; Singh, P.P.; Adholeya, A. Important innate differences in determining symbiotic responsiveness in host and non-hosts of arbuscular mycorrhiza. *Sci Rep* **2021**, *11*, 14444. https://doi.org/10.1038/s41598-021-93626-6.
158) Gaude, N.; Schulze, W.X.; Franken, P.; Krajinski, F. Cell type-specific protein and transcription profiles implicate periarbuscular membrane synthesis as an important carbon sink in the mycorrhizal symbiosis. *Plant Signal Behav* **2012**, *7*, 461-464. https://doi.org/10.4161/psb.19650.
159) Dhawi, F.; Datta, R.; Ramakrishna, W. Proteomics provides insights into biological pathways altered by plant growth promoting bacteria and arbuscular mycorrhiza in sorghum grown in marginal soil. *Biochim Biophys Acta Proteins Proteom* **2017**, *1865*, 243-251. https://doi.org/10.1016/j.bbapap.2016.11.015.
160) Sui, X.; Wu, Q.; Chang, W.; Fan, X.; Song, F. Proteomic analysis of the response of *Funneliformis mosseae/Medicago sativa* to atrazine stress. *BMC Plant Biol* **2018**, *18*, 289. https://doi.org/10.1186/s12870-018-1492-1.
161) Bai, L.; Sun, H.B.; Liang, R.T.; Cai, B.Y. iTRAQ Proteomic Analysis of Continuously Cropped Soybean Root Inoculated With *Funneliformis mosseae*. *Front Microbiol* **2019**, *10*, 61. https://doi.org/10.3389/fmicb.2019.00061.
162) Jia, T.; Wang, J.; Chang, W.; Fan, X.; Sui, X.; Song, F. Proteomics Analysis of *E. angustifolia* Seedlings Inoculated with Arbuscular Mycorrhizal Fungi under Salt Stress. *Int J Mol Sci* **2019**, *20*, 788. https://doi.org/10.3390/ijms20030788.
163) Mamontova, T.; Afonin, A.M.; Ihling, C.; Soboleva, A.; Lukasheva, E.; Sulima, A.S.; Shtark, O.Y.; Akhtemova, G.A.; Povydysh, M.N.; Sinz, A.; Frolov, A.; Zhukov, V.A.; Tikhonovich, I.A. Profiling of Seed Proteome in Pea (*Pisum sativum* L.) Lines Characterized with High and Low Responsivity to Combined Inoculation with Nodule Bacteria and Arbuscular Mycorrhizal Fungi. *Molecules* **2019**, *24*, 1603. https://doi.org/10.3390/molecules24081603.

164) Ranjbar Sistani, N.; Desalegn, G.; Kaul, H.P.; Wienkoop, S. Seed Metabolism and Pathogen Resistance Enhancement in *Pisum sativum* During Colonization of Arbuscular Mycorrhizal Fungi: An Integrative Metabolomics-Proteomics Approach. *Front Plant Sci* **2020**, *11*, 872. https://doi.org/10.3389/fpls.2020.00872.

165) Wu, J.T.; Wang, L.; Zhao, L.; Huang, X.C.; Ma, F. Arbuscular mycorrhizal fungi effect growth and photosynthesis of *Phragmites australis* (Cav.) Trin ex. Steudel under copper stress. *Plant Biol. (Stuttg)* **2020**, *22*, 62-69. https://doi.org/10.1111/plb.13039.

166) Xi, L.; Shen, Y.; Zhao, X.; Zhou, M.; Mi, Y.; Li, X.; Chen, H.; Wei, Y.; Su, H.; Hou, H. Effects of arbuscular mycorrhizal fungi on frond antimony enrichment, morphology, and proteomics in *Pteris cretica* var. nervosa during antimony phytoremediation. *Sci Total Environ* **2021**, *804*, 149904. https://doi.org/10.1016/j.scitotenv.2021.149904.

167) Gaude, N.; Bortfeld, S.; Erban, A.; Kopka, J.; Krajinski, F. Symbiosis dependent accumulation of primary metabolites in arbuscule-containing cells. *BMC Plant Biol* **2015**, *15*, 234. https://doi.org/10.1186/s12870-015-0601-7.

168) Salloum, M.S.; Insani, M.; Monteoliva, M.I.; Menduni, M.F.; Silvente, S.; Carrari, F.; Luna, C. Metabolic responses to arbuscular mycorrhizal fungi are shifted in roots of contrasting soybean genotypes. *Mycorrhiza* **2019**, *29*, 459-473. https://doi.org/10.1007/s00572-019-00909-y.

169) Bonini, P.; Rouphael, Y.; Miras-Moreno, B.; Lee, B.; Cardarelli, M.; Erice, G.; Cirino, V.; Lucini, L.; Colla, G. A Microbial-Based Biostimulant Enhances Sweet Pepper Performance by Metabolic Reprogramming of Phytohormone Profile and Secondary Metabolism. *Front Plant Sci* **2020**, *11*, 567388. https://doi.org/10.3389/fpls.2020.567388.

170) Rouphael, Y.; Lucini, L.; Miras-Moreno, B.; Colla, G.; Bonini, P.; Cardarelli, M. Metabolomic Responses of Maize Shoots and Roots Elicited by Combinatorial Seed Treatments With Microbial and Non-microbial Biostimulants. *Front Microbiol* **2020**, *11*, 664. https://doi.org/10.3389/fmicb.2020.00664.

171) Cai, X.; Zhao, H.; Liang, C.; Li, M.; Liu, R. Effects and Mechanisms of Symbiotic Microbial Combination Agents to Control Tomato *Fusarium* Crown and Root Rot Disease. *Front Microbiol* **2021**, *12*, 629793. https://doi.org/10.3389/fmicb.2021.629793.

172) Liang, S.M.; Zhang, F.; Zou, Y.N.; Kuča, K.; Wu, Q.S. Metabolomics Analysis Reveals Drought Responses of Trifoliate Orange by Arbuscular Mycorrhizal Fungi With a Focus on Terpenoid Profile. *Front. Plant Sci* **2021**, *12*, 740524. https://doi.org/10.3389/fpls.2021.740524.

173) Kurth, F.; Mailänder, S.; Bönn, M.; Feldhahn, L.; Herrmann, S.; Große, I.; Buscot, F.; Schrey, S.D.; Tarkka, M.T. *Streptomyces*-induced resistance against oak powdery mildew involves host plant responses in defense, photosynthesis, and secondary metabolism pathways. *Mol Plant Microbe Interact* **2014**, *27*, 891-900. https://doi.org/10.1094/MPMI-10-13-0296-R.

174) Dong, Z.Y.; Narsing Rao, M.P.; Wang, H.F.; Fang, B.Z.; Liu, Y.H.; Li, L.; Xiao, M.; Li, W.J. Transcriptomic analysis of two endophytes involved in enhancing salt stress ability of *Arabidopsis thaliana*. *Sci. Total Environ* **2019**, *686*, 107-117. https://doi.org/10.1016/j.scitotenv.2019.05.483.

175) Safdarian, M.; Askari, H.; Shariati, J.V.; Nematzadeh, G. Transcriptional responses of wheat roots inoculated with *Arthrobacter nitroguajacolicus* to salt stress. *Sci Rep* **2019**, *9*, 1792. https://doi.org/10.1038/s41598-018-38398-2.

176) Gong, Y.; Chen, L.J.; Pan, S.Y.; Li, X.W.; Xu, J.; Zhang, C.; Xing, K.; Qin, S. Antifungal potential evaluation and alleviation of salt stress in tomato seedlings by a halotolerant plant growth-promoting

actinomycete *Streptomyces* sp. KLBMP5084. *Rhizosphere* **2020**, 16, 100262. https://doi.org/10.1016/j.rhisph.2020.100262.

177) Belt, K.; Foley, R.C.; O'Sullivan, C.A.; Roper, M.M.; Singh, K.B.; Thatcher, L.F. A Plant Stress-Responsive Bioreporter Coupled With Transcriptomic Analysis Allows Rapid Screening for Biocontrols of Necrotrophic Fungal Pathogens. *Front Mol Biosci* **2021**, *8*, 708530. https://doi.org/10.3389/fmolb.2021.708530.

178) Gao, Y.; Ning, Q.; Yang, Y.; Liu, Y.; Niu, S.; Hu, X.; Pan, H.; Bu, Z.; Chen, N.; Guo, J.; Yu, J.; Cao, L.; Qin, P.; Xing, J.; Liu, B.; Liu, X.; Zhu, Y. Endophytic *Streptomyces hygroscopicus* OsiSh-2-Mediated Balancing between Growth and Disease Resistance in Host Rice. *mBio* **2021**, *12*, e0156621. https://doi.org/10.1128/mBio.01566-21.

179) Zhalnina, K.; Louie, K.B.; Hao, Z.; Mansoori, N.; da Rocha, U.N.; Shi, S.; Cho, H.; Karaoz, U.; Loqué, D.; Bowen, B.P.; Firestone, M.K.; Northen, T.R.; Brodie, EL. Dynamic root exudate chemistry and microbial substrate preferences drive patterns in rhizosphere microbial community assembly. *Nat Microbiol* **2018**, *3*, 470-480. https://doi.org/10.1038/s41564-018-0129-3.

180) Esmaeil Zade, N.S.; Sadeghi, A.; Moradi, P. *Streptomyces* strains alleviate water stress and increase peppermint (*Mentha piperita*) yield and essential oils. *Plant Soil* **2019**, *434*, 441-452. https://doi.org/10.1007/s11104-018-3862-8.

181) Tisserant, E.; Malbreil, M.; Kuo, A.; Kohler, A.; Symeonidi, A.; Balestrini, R.; Charron, P.; Duensing, N.; Frei dit Frey, N.; Gianinazzi-Pearson, V.; Gilbert, L.B.; Handa, Y.; Herr, J.R.; Hijri, M.; Koul, R.; Kawaguchi, M.; Krajinski, F.; Lammers, P.J.; Masclaux, F.G.; Murat, C.; Morin, E.; Ndikumana, S.; Pagni, M.; Petitpierre, D.; Requena, N.; Rosikiewicz, P.; Riley, R.; Saito, K.; San Clemente, H.; Shapiro, H.; van Tuinen, D.; Bécard, G.; Bonfante, P.; Paszkowski, U.; Shachar-Hill, Y.Y.; Tuskan, G.A.; Young, J.P.; Sanders, I.R.; Henrissat, B.; Rensing, S.A.; Grigoriev, I.V.; Corradi, N.; Roux, C.; Martin, F. Genome of an arbuscular mycorrhizal fungus provides insight into the oldest plant symbiosis. *Proc Natl Acad Sci USA* **2013**, *110*, 20117-20122. https://doi.org/10.1073/pnas.1313452110.

182) Lin, K.; Limpens, E.; Zhang, Z.; Ivanov, S.; Saunders, D.G.; Mu, D.; Pang, E.; Cao, H.; Cha, H.; Lin, T.; Zhou, Q.; Shang, Y.; Li, Y, Sharma, T.; van Velzen, R.; de Ruijter, N.; Aanen, D.K.; Win, J.; Kamoun, S.; Bisseling, T.; Geurts, R.; Huang, S. Single nucleus genome sequencing reveals high similarity among nuclei of an endomycorrhizal fungus. *PLoS Genet* **2014**, *10*, e1004078. https://doi.org/10.1371/journal.pgen.1004078.

183) Tang, N.; San Clemente, H.; Roy, S.; Bécard, G.; Zhao, B.; Roux, C. A Survey of the Gene Repertoire of *Gigaspora rosea* Unravels Conserved Features among Glomeromycota for Obligate Biotrophy. *Front Microbiol* **2016**, *7*, 233. https://doi.org/10.3389/fmicb.2016.00233.

184) Beaudet, D.; Chen, E.C.H.; Mathieu, S.; Yildirir, G.; Ndikumana, S.; Dalpé, Y.; Séguin, S.; Farinelli, L.; Stajich, J.E.; Corradi, N. Ultra-low input transcriptomics reveal the spore functional content and phylogenetic affiliations of poorly studied arbuscular mycorrhizal fungi. *DNA Res* **2018**, *25*, 217-227. https://doi.org/10.1093/dnares/dsx051.

185) Chen, E.C.H.; Morin, E.; Beaudet, D.; Noel, J.; Yildirir, G.; Ndikumana, S.; Charron, P.; St-Onge, C.; Giorgi, J.; Krüger, M.; Marton, T.; Ropars, J.; Grigoriev, I.V.; Hainaut, M.; Henrissat, B.; Roux, C.; Martin, F.; Corradi, N. High intraspecific genome diversity in the model arbuscular mycorrhizal symbiont *Rhizophagus irregularis*. *New Phytol* **2018**, *220*, 1161-1171. https://doi.org/10.1111/nph.14989.

186) Sun, X.; Chen, W.; Ivanov, S.; MacLean, A.M.; Wight, H.; Ramaraj, T.; Mudge, J.; Harrison, M.J.; Fei, Z. Genome and evolution of the arbuscular mycorrhizal fungus *Diversispora epigaea* (formerly *Glomus*

*versiforme*) and its bacterial endosymbionts. *New Phytol* **2019**, *221*, 1556-1573. https://doi.org/10.1111/nph.15472.

187) Morin, E.; Miyauchi, S.; San Clemente, H.; Chen, E.C.H.; Pelin, A.; de la Providencia, I.; Ndikumana, S.; Beaudet, D.; Hainaut, M.; Drula, E.; Kuo, A.; Tang, N.; Roy, S.; Viala, J.; Henrissat, B.; Grigoriev, I.V.; Corradi, N.; Roux, C.; Martin, F.M. Comparative genomics of *Rhizophagus irregularis, R. cerebriforme*, *R. diaphanus* and *Gigaspora rosea* highlights specific genetic features in Glomeromycotina. *New Phytol* **2019**, *222*, 1584- 1598. doi: 10.1111/nph.15687.

188) Venice, F.; Ghignone, S.; Salvioli di Fossalunga, A.; Amselem, J.; Novero, M.; Xianan, X.; Sędzielewska Toro, K.; Morin, E.; Lipzen, A.; Grigoriev, I.V.; Henrissat, B.; Martin, F.M.; Bonfante, P. At the nexus of three kingdoms: the genome of the mycorrhizal fungus *Gigaspora margarita* provides insights into plant, endobacterial and fungal interactions. *Environ Microbiol* **2020**, *22*, 122-141. https://doi.org/10.1111/1462-2920.14827.

189) Tocchetti, A.; Bordoni, R.; Gallo, G.; Petiti, L.; Corti, G.; Alt, S.; Cruz, J.C.; Salzano, A.M.; Scaloni, A.; Puglia, A.M.; De Bellis, G.; Peano, C.; Donadio, S.; Sosio, M. A Genomic, Transcriptomic and Proteomic Look at the GE2270 Producer *Planobispora rosea*, an Uncommon Actinomycete. *PLoS One* **2015**, *10*, e0133705. https://doi.org/10.1371/journal.pone.0133705.

190) Han, J.; Gao, Q.X.; Zhang, Y.G.; Li, L.; Mohamad, O.A.A.; Rao, M.P.N.; Xiao, M.; Hozzein, W.N.; Alkhalifah, D.H.M.; Tao, Y.; Li, W.J. Transcriptomic and Ectoine Analysis of Halotolerant *Nocardiopsis gilva* YIM 90087T Under Salt Stress. *Front Microbiol* **2018**, *9*, 618. https://doi.org/10.3389/fmicb.2018.00618.

191) Liu, C.; Fan, D.; Li, Y.; Chen, Y.; Huang, L.; Yan, X. Transcriptome analysis of *Valsa mali* reveals its response mechanism to the biocontrol actinomycete *Saccharothrix yanglingensis* Hhs.015. *BMC Microbiol* **2018**, *18*, 90. https://doi.org/10.1186/s12866-018-1225-5.

192) Wang, C.; Chen, Y.; Zhou, H.; Li, X.; Tan, Z. Adaptation mechanisms of *Rhodococcus* sp. CNS16 under different temperature gradients: Physiological and transcriptome. *Chemosphere* **2020**, *J238*, 124571. https://doi.org/10.1016/j.chemosphere.2019.124571.

193) Zhang, Y.; Li, Y.; Zhang, Y.; Wang, Z.; Zhao, M.; Su, N.; Zhang, T.; Chen, L.; Wei, W.; Luo, J.; Zhou, Y.; Xu,Y.; Xu, P., Li, W.; Tao, Y. Quantitative Proteomics Reveals Membrane Protein-Mediated Hypersaline Sensitivity and Adaptation in Halophilic *Nocardiopsis xinjiangensis*. *J Proteome Res* **2016**, *15*, 68-85. https://doi.org/10.1021/acs.jproteome.5b00526.

194) Lee, M.Y.; Kim, H.Y.; Lee, S.; Kim, J.G.; Suh, J.W.; Lee, C.H. Metabolomics-Based Chemotaxonomic Classification of *Streptomyces* spp. and Its Correlation with Antibacterial Activity. *J Microbiol Biotechnol* **2015**, *25*, 1265-1274. https://doi.org/10.4014/jmb.1503.03005.

195) Takehana, Y.; Umekita, M.; Hatano, M.; Kato, C.; Sawa, R.; Igarashi, M. Fradiamine A, a new siderophore from the deep-sea actinomycete *Streptomyces fradiae* MM456M-mF7. *J Antibiot (Tokyo)* **2017** 70, 611-615. https://doi.org/10.1038/ja.2017.26.

196) Elmahdy, M.H.; Azmy, A.F.; El-Gebaly, E.; Saafan, A.; Gaber, Y. A Comparative Proteomic Study of *Thermobifida cellulosilytica* TB100T Secretome Grown on Carboxymethylcellulose and Rice Straw. *Open Biotechnol J* **2020**, *14*, 42-51. https://doi.org/10.2174/1874070702014010042.

197) Armin, R.; Zühlke, S.; Grunewaldt-Stöcker, G.; Mahnkopp-Dirks, F.; Kusari, S. Production of Siderophores by an Apple Root-Associated *Streptomyces ciscaucasicus* Strain GS2 Using Chemical and Biological OSMAC Approaches. *Molecules* **2021**, *26*, 3517. https://doi.org/10.3390/molecules26123517.
